# Delayed Auditory Feedback and Transcranial Direct Current Stimulation Treatment for the Enhancement of Speech Fluency in Adults Who Stutter: Protocol for a Randomized Controlled Trial

**DOI:** 10.2196/16646

**Published:** 2020-04-21

**Authors:** Narges Moein, Reyhane Mohamadi, Reza Rostami, Michael Nitsche, Reza Zomorrodi, Amir Ostadi, Abbasali Keshtkar

**Affiliations:** 1 Department of Speech and Language Pathology School of Rehabilitation Sciences Iran University of Medical Sciences Tehran Iran; 2 Rehabilitation Research Center Iran University of Medical Sciences Tehran Iran; 3 Faculty of Psychology University of Tehran Tehran Iran; 4 Department of Psychology and Neurosciences Leibniz Research Centre for Working Environment and Human Factors Dortmund Germany; 5 Temerty Centre for Therapeutic Brain Intervention, Centre for Addiction and Mental Health Department of Psychiatry University of Toronto Toronto, ON Canada; 6 Faculty of Science University of Waterloo Waterloo, ON Canada; 7 Department of Health Sciences Education Development School of Public Health Tehran University of Medical Sciences Tehran Iran

**Keywords:** delayed auditory feedback, stuttering, transcranial direct current stimulation, speech fluency

## Abstract

**Background:**

Stuttering is a complex speech disorder that affects speech fluency. Recently, it has been shown that noninvasive brain stimulation may be useful to enhance the results of fluency interventions in adults who stutter. Delayed auditory feedback (DAF) is a method to enhance speech fluency in individuals who stutter. Adjunctive interventions are warranted to enhance the efficacy of this intervention.

**Objective:**

Individuals who stutter have pathological activation patterns in the primary and secondary auditory areas. Consequently, in this study, we hypothesize that stimulation of these areas might be promising as an adjunctive method to fluency training via DAF to enhance speech therapy success in individuals with a stutter. We will systematically test this hypothesis in this study.

**Methods:**

This study is designed as a randomized, double-blind, sham-controlled clinical trial. All participants will receive DAF. The intervention group will additionally receive real transcranial direct current stimulation, while the control group will be exposed to sham stimulation. The assignment of the participants to one of these groups will be randomized. Before starting the treatment program, 2 preintervention assessments will be conducted to determine the severity of stuttering. Once these assessments are completed, each subject will participate in 6 intervention sessions. Postintervention assessments will be carried out immediately and 1 week after the last intervention session. Subsequently, to explore the long-term stability of the treatment results, the outcome parameters will be obtained in follow-up assessments 6 weeks after the treatment. The primary outcome measurement—the percentage of stuttered syllables—will be calculated in pre-, post-, and follow-up assessments; the secondary outcomes will be the scores of the following questionnaires: the Stuttering Severity Instrument–Fourth Edition and the Overall Assessment of the Speaker’s Experience of Stuttering.

**Results:**

This protocol was funded in 2019 and approved by the Research Ethics Committee of the Iran University of Medical Sciences in June 2019. Data collection started in October 2019. As of February 2020, we have enrolled 30 participants. We expect data analysis to be completed in April 2020, and results will be published in summer 2020.

**Conclusions:**

We anticipate that this study will show an adjunctive effect of transcranial direct current stimulation, when combined with DAF, on stuttering. This should include not only a reduction in the percentage of stuttered syllables but also improved physical behavior and quality of life in adults who stutter.

**Trial Registration:**

ClinicalTrial.gov NCT03990168; https://clinicaltrials.gov/ct2/show/NCT03990168

**International Registered Report Identifier (IRRID):**

DERR1-10.2196/16646

## Introduction

Stuttering is a complex speech disorder that affects speech fluency, as defined by repetitions, prolongations, and blocks in speech sound [1]. It is prevalent in 1% of the population [2]. Despite advances in the treatment of stuttering, major limitations such as instability of treatment outcomes and lack of long-term results have yet to be addressed [3]. Recently, it has been shown that noninvasive brain stimulation may be useful to enhance the results of fluency interventions in adults who stutter [4]. One of these tools is transcranial direct current stimulation (tDCS), a noninvasive brain stimulation technique that uses a weak, constant current of 1-2 mA that passes through the scalp and results in alterations in cortical excitability and activity [5].

Structural and functional neuroimaging in individuals who stutter show bilateral underactivation of the primary and secondary auditory areas as compared to adults who do not stutter [6]. Delayed auditory feedback (DAF) is a method to enhance speech fluency in individuals who stutter. In this method, alteration of the timing feedback affects speech rate and consequently results in increased fluency [7]. A recent study has shown that in individuals who undergo DAF, enhanced speech fluency is associated with increased activity in the primary and secondary auditory areas of the superior temporal gyrus [8]. DAF is, however, time-consuming, and long-term reductions in stuttering are still restricted [9]. Adjunctive interventions are thus warranted to enhance the efficacy of this intervention. Thus, to increase speech fluency, we will combine DAF as a speech fluency intervention with tDCS applied over the superior temporal gyrus (electrode position T3 of the 10-20 international system) to enhance efficacy of the fluency intervention. Given past evidence that tDCS is capable of inducing plasticity-like changes in cortical functions that can outlast the stimulation period, we anticipate that tDCS will help stabilize intervention-related improvements in speech fluency [10].

In tDCS, electrical current flows between 2 or more electrodes—a positively charged anode and a negatively charged cathode—which are positioned at specified locations on the scalp. The current produced by tDCS results in subtle changes in the resting membrane potential of cortical neurons in the underlying brain tissue [5]. Specifically, with standard protocols, changes under the anode (referred to as anodal stimulation) result in the depolarization of critical neuronal compartments, thus increasing neuronal excitability, while changes under the cathode (cathodal stimulation) result in the hyperpolarization of respective compartments and decrease excitability at the macroscopic level [11]. Neuroplastic effects emerge after some minutes of stimulation, depending on alterations to glutamatergic and GABAergic activities [12,13]. Similar to the acute membrane polarization effects, anodal tDCS and cathodal tDCS result in excitability-enhancing and excitability-reducing plasticity, respectively [10,14,15].

In recent years, studies have shown that tDCS enhances speech fluency in adults who stutter when applied during a fluency intervention [4,16]. Since, in addition to the temporal auditory areas, the frontal cortex shows abnormalities during stuttering, tDCS was applied over the latter area in a recent study [4]. Chesters et al [4] investigated the effect of 5 sessions of anodal tDCS over the left inferior frontal gyrus during a speech fluency intervention on stuttering. Speech fluency significantly improved in the treatment group that received anodal tDCS combined with the fluency intervention in comparison with the respective sham tDCS group. It was concluded that using tDCS simultaneously with fluency training can enhance speech fluency in adults who stutter.

Due to impaired sensory-motor integration, stutterers have pathological activation patterns in the temporal lobe (ie, the primary and secondary auditory areas are underactive while speaking) [6]. Consequently, in this study, we hypothesize that stimulation of these areas might be promising as an adjunctive method to fluency training via DAF, to enhance speech therapy success in individuals with a stutter. We will systematically test this hypothesis in this study.

## Methods

### Overview

This project aims to investigate the effect of adjunctive noninvasive brain stimulation on speech fluency. To this aim, we will recruit 2 groups of participants—an intervention group and a control group. In the intervention group, participants will receive anodal tDCS simultaneously with DAF as a fluency intervention, whereas the participants in the control group will receive sham stimulation during DAF. The population of this study will be adults with a stutter. We hypothesize that anodal tDCS over the temporal target area will improve the efficacy of DAF to enhance speech fluency and will stabilize treatment effects.

### Hypothesis

We hypothesize that the efficacy of the intervention to enhance fluency of speech in individuals with a stutter is improved when combined with anodal tDCS over the superior temporal gyrus, as compared to a sham tDCS control condition.

### Primary Objectives

The primary objective of this proposal is to compare the mean score of the percentage of stuttered syllables (SS%) [17] between the intervention and control groups at 5 time points: 1 week before treatment; immediately before treatment; and immediately, 1 week, and 6 weeks after treatment.

### Secondary Objectives

The secondary objectives are as follows:

- Comparison of the mean score of stuttered syllables, duration, physical concomitant behaviors, and total score of the Stuttering Severity Instrument-Fourth Edition (SSI-4) questionnaire [18] between the intervention and control groups 1 week before treatment; immediately before treatment; and immediately, 1 week, and 6 weeks after treatment.
- Comparison of the means of the general information section, reaction to stuttering section, communication in daily situations section, quality of life section, total impact score, and impact rating of the Overall Assessment of the Speaker’s Experience of Stuttering (OASES) questionnaire [19] between the intervention and control groups 1 week before treatment; immediately before treatment; and immediately, 1 week, and 6 weeks after treatment.

### Efficacy

We expect adjunctive tDCS to enhance the magnitude of the effects of the intervention on our outcome parameters and to enhance the stability of improvements made due to the treatment.

### Safety

There is no anticipated relevant risk associated with the stimulation parameters used in this study [20]. In a systematic review evaluating adverse effects of all published tDCS studies, including studies conducted in vulnerable populations, adverse effects of tDCS such as tingling and itching sensations under the electrodes were mild and disappeared soon after stimulation. No severe adverse effects of tDCS have been documented so far. It thus can be concluded that tDCS within the proposed stimulation parameters is safe [21].

### Study Population

The study population will include adults with moderate-to-severe stuttering who have not received any treatment at least 1 month prior to the intervention. A speech-language pathologist will assess the severity of stuttering in each subject to determine whether he or she can be enrolled. The SSI-4 questionnaire will be used to assess stuttering severity. Inclusion, exclusion, and withdrawal criteria are listed in Textbox 1.

**Inclusion, exclusion, and withdrawal criteria.**

**Inclusion criteria:**

- History of developmental stuttering
- Participants diagnosed with moderate-to-severe stuttering
- Right-handed
- 18 to 50 years of age (adult)
- Native speaker of Farsi
- Nonsmoker

**Exclusion criteria:**

- Stuttering accompanied by other speech or language disorders
- Stuttering treatment within 1 month prior to the intervention
- Hearing loss
- History of neurological or psychiatric disorders
- History of seizures
- Intake of any medication that affects brain functions (eg, antidepressants)
- Pregnancy
- Breastfeeding
- Cranial bone defects
- Cranial or brain metal implants
- Skin lesions

**Withdrawal criteria:**

- Request to withdraw from the study at any point during the treatment program
- Skin damage or major adverse effects of stimulation

### Study Design

This study is designed as a randomized, double-blind, sham-controlled clinical trial. Participants will be randomly allocated to a control or intervention group. All participants will receive DAF during the intervention. The intervention group will additionally receive anodal tDCS, while the control group is exposed to sham tDCS. Data will be collected via questionnaires, voice recordings, and observations at 5 time points (ie, 1 week and immediately before the intervention; and immediately, 1 week, and 6 weeks post intervention). By comparing the outcomes of the respective treatments, we will investigate the efficacy of anodal tDCS combined with DAF in enhancing speech fluency.

Before starting the treatment program, 2 preintervention assessments will be conducted to determine the severity of stuttering. Once these assessments are completed, each subject will participate in 6 intervention sessions. Postintervention assessments will be carried out immediately and 1 week after the last intervention session. Afterward, to explore the long-term stability of the treatment results, the outcome parameters will be obtained in follow-up assessments 6 weeks after the treatment. This single-center trial will be conducted at the Vahdat Neurorehabilitation Clinic in Tehran, Iran.

As previously mentioned, the study will be conducted on adults with moderate-to-severe stuttering; hence, prior to recruitment, the severity of stuttering will be assessed to determine enrollment eligibility. In order to determine the severity of stuttering, an SSI-4 score for each individual will be calculated.

We will obtain two baseline measurements—one at 1 week and the other immediately before the intervention—to guarantee symptom stability. At baseline, the participants will be visited individually, and their voices will be recorded while performing 3 different tasks (ie, oral reading, monologue, and conversation). Based on these data, we will calculate the primary outcome measure (ie, SS%). The voice of the participants will be recorded by a H5 Handy Recorder ZOOM). Two experienced specialist raters will independently count the SS% of all speech samples. In order to determine interrater reliability, Cohen kappa will be calculated. In cases of agreement between the raters (intraclass correlation coefficient=0.8-1), the SS% will be reported. In cases of disagreement (intraclass correlation coefficient <0.8), a third senior rater will evaluate SS%.

During the two baseline sessions, the principal investigator will observe physical behavior, which is required to calculate the SSI-4 score. The final part of the preintervention assessments is the completion of the OASES questionnaire by the participants. After the second baseline assessment, the intervention will start and carry on for 6 consecutive days.

**Figure 1:**
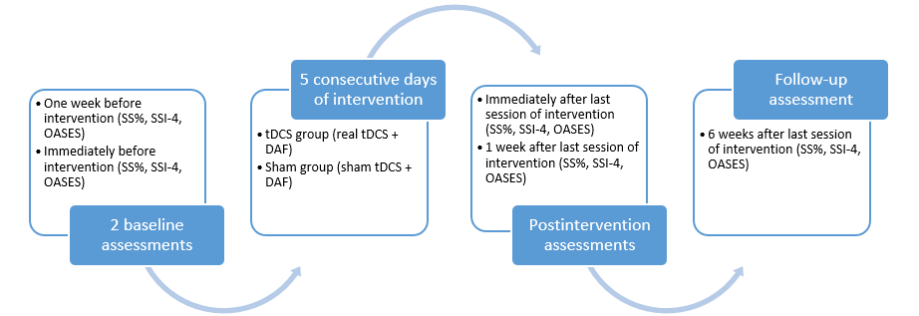
Trial design and timeline. DAF: Delayed Auditory Feedback; OASES: Overall Assessment of the Speaker’s Experience of Stuttering; SS%: percentage of stuttered syllables; SSI-4: Stuttering Severity Instrument-Fourth Edition; tDCS: transcranial direct current stimulation.

At 3 time points after the last intervention session (ie, immediately, 1 week, and 6 weeks), each subject will undergo postintervention assessments, which will be identical to the preintervention assessments and obtained by the same raters. The trial design and timeline are shown in Figure 1.

After each intervention session, participants will fill out a questionnaire using a 5-point Likert scale (1=very mild, 5=very severe) to report potential side effects of tDCS, including itching, burning, tingling, headache, fatigue, sudden mood change, difficulties in concentration, changes in visual perception, unpleasant somatosensory sensations, unpleasant visual sensations, nausea, drowsiness, persisting feelings of stimulation, and 1 open question for any other adverse effects.

In addition, to ensure successful blinding, participants will be asked to guess the type of treatment they will or had received (ie, anodal tDCS or sham) before and after the intervention.

### Randomization and Blinding

This study is a randomized, double-blind, sham-controlled trial. Participants will be randomly allocated to the intervention and control groups. The random assignment of participants ensures the prevention of a possible selection bias and a disbalance of confounding factors between the study arms. Randomization will be performed via a web-based randomization tool [22]. In accordance with the requirements of this tool, we will include 50 subjects and 4 blocks of equal size, for which randomization will be performed independently. Each subject will be given an ID number and will be assigned to one of the treatment groups. The individual responsible for generating the random list will not be involved in any other part of the trial.

As the study is double-blinded, neither the participants nor the investigators will know which group each participant will be assigned to. Sealed opaque envelopes will be used for concealment. Accordingly, an envelope will be produced for each subject. These envelopes will be marked with an ID number, and the treatment group associated with each ID will be placed in its respective envelope. In order to guarantee double blinding, specific letters, unknown to the principal investigator and the subjects, will be used instead of the actual names of the treatment groups (ie, intervention and control) inside the envelopes. Assignment of these letters to the respective intervention group will be done by a clinician (not the principal investigator). Before the principal investigator starts the treatment of the respective participant, the subject will be given one of the sealed envelopes by the clinician. Once the envelope is opened by the corresponding participant, the clinician will set up the mode—anodal or sham—based on the content of the subject's envelope and attach the electrodes to the participant´s head as illustrated in the sections below. Once completed, the principal investigator will commence the treatment. The researchers who are responsible for outcome assessment and data analysis will also be blinded to the intervention groups.

### Intervention Program

#### Performance of Transcranial Current Direct Stimulation

The stimulation will be done by passing a current of 1 mA between two 5 cm × 7 cm electrodes for a duration of 20 minutes with ramp-up and ramp-down intervals of 15 seconds. This common setup has been shown to be efficient for enhancing speech fluency in previous tDCS studies [3,4]. A neuroConn DC-STIMULATOR will be used to deliver tDCS. The electrodes used for tDCS are conductive rubber electrodes encased in a sponge pocket, and saline solution will be used as an electrolyte-based contact medium. The electrode sponges must be saturated with saline. It is crucial that each side of the sponges is sufficiently moisturized, but not overly saturated, to avoid dripping. Before placing electrodes on the scalp, the clinician will inspect the skin for any skin damage or lesion. For both anodal and sham modes, the same stimulation intensity parameters will be used. However, for the sham stimulation, the device will ramp down automatically after 30 seconds. The intervention will be conducted for 6 consecutive days.

**Figure 2:**
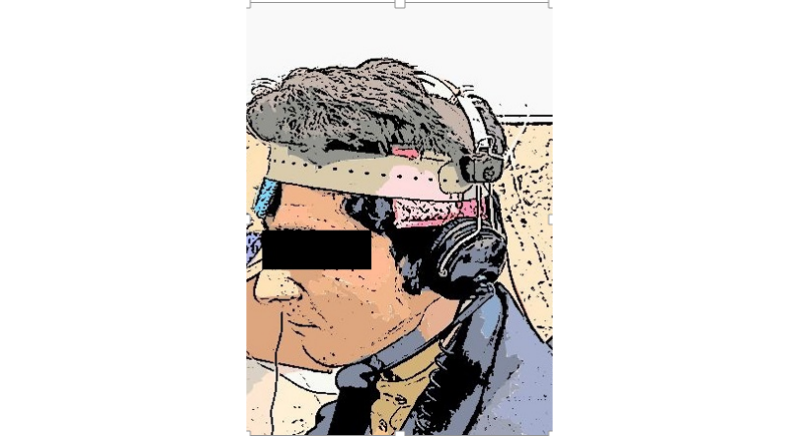
Electrode placement.

An electroencephalogram cap will be used to identify the site of stimulation. The anode electrode will be placed over the left superior temporal gyrus (T3 according to the 10-20 international system [23]), and the cathode electrode will be positioned over the right frontopolar region (Fp2 according to the 10-20 system [23]). To prevent movement of the electrodes, their positions will be fixed by elastic rubber straps. Electrode placement is shown in Figure 2.

#### Performance of Delayed Auditory Feedback

During stimulation, participants of both groups will receive DAF as a fluency treatment. In order to deliver DAF, Audapter, which is a software package for manipulating the acoustic parameters of speech in real time, will be used [24]. This package consists of the core algorithm for real-time manipulation and a MATLAB wrap-around. The real-time signal processing algorithms are coded in C++. The subjects will perform 3 tasks (ie, oral reading, monologue, and conversation) with DAF <60 ms. This 60-ms delay has been shown to be efficient to enhance speech fluency in individuals with a stutter [25].

Although, as outlined above, we do not anticipate major adverse events, the occurrence of skin irritations, itching, tingling, burning, or pain (including headache) will be monitored. After each intervention session, a side effects questionnaire will be filled out by participants. Furthermore, participants will guess if they received anodal or sham stimulation before the intervention, after the first session, and after the intervention period to ensure successful blinding.

Data from the respective questionnaires will be recorded on paper, and recordings of subjects’ voices will be stored electronically. Access to data resources will be restricted to the investigators. Finally, all study documents will be securely maintained for 2 years.

### Statistical and Analytical Analyses

In this proposal, the null hypothesis (H_0_) is that the combined treatment method using tDCS stimulation and DAF, as compared to DAF combined with sham stimulation, has no additional effect on the enhancement of speech fluency in adults who stutter.

#### Intended Sample Size

Based on the previous study by Chester et al [4], and using G*Power software [26], 25 subjects per group (50 participants in total) are required to detect a significant difference for a time × group interaction using a two-way mixed model analysis of variance (ANOVA). Time is the within-subject factor (ie, before the intervention and immediately, 1 week, and 6 weeks after the intervention), and group is the between-subject factor (ie, anodal or sham tDCS). The dependent variable is stuttered syllables (SS%). Type I (α) and type II (β) errors are set at .05 and .20, respectively, and the effect size is 0.17.

#### Other Statistical Analyses

Descriptive statistics will be calculated for demographic and baseline characteristics as well as primary and secondary outcomes for all participants. For every subject, summary statistics including mean, SD, median, minimum, and maximum will be provided for quantitative variables. For qualitative variables, frequency tables will be presented. Group differences of demographic variables will be assessed by Student *t* tests for quantitative variables and chi-square tests for qualitative variables.

In order to evaluate the effect of the treatment, the early and late outcomes (ie, measurements taken immediately, 1 week after treatment, and 6 weeks after treatment) will be examined. Depending on the significance of the ANOVA results, exploratory post hoc Student *t* tests will be conducted to compare conditions. The same procedures will be conducted for the secondary outcome parameters.

Data on tolerability and safety will be analyzed using an independent samples *t* test for common adverse events. Reports of rare side effects will be documented. To confirm successful blinding, a chi-square test will be applied.

Participants may be lost to follow-up for two reasons: (1) they have not completed at least 80% of the treatment (5 out of 6 sessions) and (2) they did not show up for the outcome measurement sessions 1 week or 6 weeks after the intervention. In these cases, intention-to-treat and modified intention-to-treat approaches will be employed. For cases lost to follow-up, efforts will be made to identify the reason, for example, by phone.

### Ethics Approval and Consent to Participate

This study will be carried out in accordance to the ethical principles and national norms and standards for conducting medical research in Iran (approval ID: IR.IUMS.REC.1398.352, Iran National Committee for Ethics in Biomedical Research). In addition, our informed consent form will be reviewed and approved by the ethics committee. Prior to the commencement of any part of the study, the principal investigator will obtain written informed consent from all participants.

### Consent for Publication

All study findings and information will be posted on ClinicalTrials.gov (identifier NCT03990168). Although the investigator will be free to use the study findings for educational and scientific purposes, written consent will be obtained from the study sponsor prior to submission of any manuscript for publication.

### Availability of Data and Materials

Information about subjects will be kept confidential and will not be available for public access. In addition, any document, data, voice recording, and other records will be identified by a participant ID number, and the name of each subject will be kept confidential. The data sets used and analyzed during the study are available from the corresponding author upon reasonable request.

## Results

This protocol was funded in 2019 and approved by the Research Ethics Committee of the Iran University of Medical Sciences in June 2019. Data collection started in October 2019. As of February 2020, we have enrolled 30 participants. We expect that data analysis will be completed in April 2020, and the results will be published in summer 2020.

## Discussion

This is the first randomized controlled trial with follow-up measures to explore the efficacy of tDCS combined with DAF to improve speech fluency in adults who stutter. In this study, the impact of the combined treatment will be evaluated by SS% as the primary outcome measure. Treatment impact will be examined for up to 6 weeks to exclude any temporary effects with limited clinical value [1]. The findings of this study will thus supply information about the efficacy of tDCS as an adjunctive therapy for reducing SS% and its stability over a prolonged time period.

In addition, we will obtain the SSI-4 score before and after treatment. This score includes additional information such as changes in the duration of stutter moments and physical concomitants of stuttering. These parameters disrupt speech fluency and have negative effects on communication. Reduction in these behaviors, as expected, are relevant for the enhancement of communication efficacy in adults who stutter [18].

Finally, we will survey the impact of stuttering on a person’s quality of life by using the OASES questionnaire. As a comprehensive assessment of stuttering, this questionnaire measures the effect of stuttering on multiple life situations [19].

In summary, we anticipate that this study will show an adjunctive effect of tDCS, when combined with DAF, on stuttering. This should include not only a reduction in SS% but also improved physical behavior and quality of life in adults who stutter.

## Appendix

Multimedia Appendix 1 Peer review comments from the Iran University of Medical Sciences.

## Abbreviations

ANOVA: analysis of variance
DAF: delayed auditory feedback
OASES: Overall Assessment of the Speaker’s Experience of Stuttering
SS%: percentage of stuttered syllables
SSI-4: Stuttering Severity Instrument-Fourth Edition
tDCS: transcranial direct current stimulation
