# Supplementary material for: Delayed Auditory Feedback and Transcranial Direct Current Stimulation Treatment for the Enhancement of Speech Fluency in Adults Who Stutter: Protocol for a Randomized Controlled Trial
Source: JMIR Res Protoc. 2020 Apr 21;9(4):e16646. doi: 10.2196/16646 (PMC7201314; doi:10.2196/16646)
Supplement: Multimedia Appendix 1 [file resprot_v9i4e16646_app1.pdf]

فرم اعلام اشکالات پروپوزال پایان‌نامه دکتری

دانشجو: خانم نرگس معین

استاد راهنما: دکتر محمدی و دکتر رستمی

استاد داور: دکتر امیری‌شوکی

درج اصلاحات پیشنهادی استاد محترم داور	پاسخ دانشجو قابل قبول است.		ملاحظات
	بلی	خیر	
با سلام و عرض ادب<br>و تبریک و آرزوی موفقیت برای جنابعالی<br>لطفا اشکالات نگارشی اصلاح شود. مثلا تورفتگی اول پاراگراف<br>متغیر اول "تحریک مغز"، آیا واقعا متغیر است؟<br>در خصوص جدول متغیرها نظراتی داشتم که چون آقای دکتر کمالی فرمودند و یادداشت فرمودید و بنده نمی‌نویسم.			
آیا با DAF، مغز را تحریک می کنید؟ منظورتان چیست؟	*		
دلیل استفاده از DAF؟ آیا به تنهایی درمان قابل قبولی هست یا چون نمی خواهند درمان شوند ....؟<br>با حذف ERP موافقم.	*		
اثر placebo را چه کار می کنید؟ چرا گروه tdcs به تنهایی ندارید؟<br>مقایسه دو گروه را اضافه بفرمایید.<br>جدول صفحه ۱۸ و ۱۹ یکجا باشد.<br>منابع را انگلیسی پاراگراف‌بندی نمایید.	*		

نام داور:

امضا:

جزئیات طرح پایان نامه

۱-۵: مقدمه و بیان مسئله

طبق تعریف سازمان بهداشت جهانی[1] (WHO)، لکنت اختلال در ریتم گفتار است که با تکرار، کشیده‌گویی و قفل‌های غیرارادی در گفتار مشخص می‌شود. شیوع لکنت ۵٪ در کودکان و ۱٪ در افراد بزرگسال است [۱]. لکنت یک اختلال پیچیده و نامتناجس[2] است [۲] و به همین دلیل تئوری‌ها، مدل‌ها و فرضیات مختلفی در مورد علت لکنت مطرح شده‌است. از جمله می‌توان به مشکل در برتری طرفی مغز [۳]، عدم تطابق بین پیچیدگی گفتار و مهارتهای حرکتی در حین رشد [۴, ۵]، مشکلات روانشناختی [۶, ۷] و مشکل در هسته‌های قاعده‌ای [۸, ۹] اشاره نمود. یکی دیگر از نظریه‌هایی که در دهه‌های اخیر به تبیین علت زیر بنایی لکنت می پردازد تئوری یکپارچگی حسی حرکتی است. بر اساس این نظریه علت زیر بنایی لکنت مشکل در یکپارچگی حسی حرکتی[3] تولید گفتار می‌باشد [۱۰, ۱۱]. مدلی که براساس این نظریه در مورد لکنت مطرح شده است مدل دیوا[4] (DIVA) نام دارد. با توجه به تبیین دقیقی که مدل دیوا در مورد چگونگی به وجود آمدن لکنت ارائه می دهد در ادامه شرح مختصری بر اجزای این مدل و علت ایجاد لکنت براساس این مدل ارائه می‌دهیم. در مدل DIVA تعاملات حسی-حرکتی درگیر در کنترل اندام‌های گویایی در حین تولید گفتار توضیح داده می‌شود. در آخرین نسخه این مدل (۲۰۱۰) که لکنت بر اساس آن تبیین شده است سه زیر سیستم وجود دارد: ۱-زیرسیستم کنترل فیدبکی[5]، ۲- زیرسیستم کنترل فیدفورواردی[6]، و ۳- زیرسیستم نظارت[7] (پایش). در این مدل زیر سیستم کنترل فیدبکی به دو بخش تقسیم می شود ۱- زیرسیستم کنترل فیدبک شنیداری ۲- زیر سیستم کنترل فیدبک سوماتوسنسوری [۱۲]. به طور کلی در مدل دیوا تولید یک واج یا هجا با فعالیت سلولهای speech sound map آغاز می‌شود، که فرض می‌شود در کورتکس پیش حرکتی شکمی[8] قرار دارند. ناحیه‌ای که گاهی Frontal operculum هم نامیده می‌شود. پس از فعال شدن speech sound map، دستورات حرکتی از طریق دو زیر سیستم کنترلی فیدبکی و فیدفورواردی به کورتکس حرکتی می‌رسند. در سیستم کنترل فیدبکی سیگنالها از کورتکس پیش حرکتی به کورتکس حسی حرکتی و شنیداری منتقل می‌شوند و دستورات حسی برای صدا را رمزگذاری می‌نمایند. پروجکشن سیناپسی دیگری نیز از speech sound map به کورتکس حرکتی مدل می‌روند و دستورات حرکتی فیدفوروارد را می‌دهند. طی تولید صدا، نواحی حرکتی هدف با وضعیت شنیداری حال حاضر مقایسه می‌شود و هرگونه ناهمخوانی بین هدف و شکل وضعیت موجود، که خطای شنیداری[9] نامیده می‌شود، منجر به صدور فرمان به کورتکس حرکتی برای تصحیح این ناهمخوانی (از طریق پروجکشن از مناطق کورتکس شنیداری به کورتکس حرکتی) می‌شود. در اولین تلاش برای تولید، مدل دستورات فیدفورواردی تنظیم شده‌ای برای صدا ندارد، بنابراین ممکن است بر زیرسیستم کنترل فیدبک شنیداری تاکید بسیار داشته باشد. در هر تلاش برای تولید صدا، دستورات فیدفورواردی به روزرسانی می‌شود تا دستوراتی که توسط زیر سیستم کنترل فیدبک شنیداری در آن تلاش ایجاد می‌شود را ترکیب کند و این کار منجر به دستورات فیدفوردارد‌ی صحیح‌تری می‌شود. سرانجام دستور فیدفورواردی خودش برای تولید صدا در وضعیت طبیعی، کافی است. دستور فیدفورواردی به اندازه کافی دقیق است و درنتیجه تعداد بسیار کمی خطای شنیداری طی تولید صدا ایجاد می‌نماید و بنابراین دیگر زیرسیستم کنترل فیدبکی را به کمک فرا نمی‌خواند. در این مرحله مدل می‌تواند صداهای گفتاری را به صورت روان تولید کند [۱۳]. فرضیه مدل دیوا در مورد لکنت این است که افراد مبتلا به لکنت نسبت به افراد غیرلکنتی تکیه بیش از اندازه بر فیدبک حسی دارند. در نتیجه آسیب به سیستم کنترل فیدفورواردی افراد مبتلا به لکنت بیشتر بر استراتژی کنترل حرکتی بر پایه فیدبک تکیه می‌کنند. چنین

---

1 World Health Organization
2 Heterogeneous
3 Sensorimotor integration
4 Directions into velocities of articulators
5 Feedback control subsystem
6 Feedforward control subsystem
7 Monitoring subsystem
8 Ventral premotor cortex
9 Auditory Error

بهتر است برای مدل دیوا ترجمه abbriviation آن گفته شود. این مدل توسط چه کسی یا افرادی طراحی شده است؟

متن بیان مسئله خیلی پیچیده نوشته شده است!

جزئیات طرح پایان نامه

۱-۵: مقدمه و بیان مسئله

طبق تعریف سازمان بهداشت جهانی[1] (WHO)، لکنت اختلال در ریتم گفتار است که با تکرار، کشیده‌گویی و قفل‌های غیرارادی در گفتار مشخص می‌شود. شیوع لکنت ۵٪ در کودکان و ۱٪ در افراد بزرگسال است [۱]. لکنت یک اختلال پیچیده و نامتناجس[2] است [۲] و به همین دلیل تئوری‌ها، مدل‌ها و فرضیات مختلفی در مورد علت لکنت مطرح شده‌است. از جمله می‌توان به مشکل در برتری طرفی مغز [۳]، عدم تطابق بین پیچیدگی گفتار و مهارتهای حرکتی در حین رشد [۴, ۵]، مشکلات روانشناختی [۶, ۷] و مشکل در هسته‌های قاعده‌ای [۸, ۹] اشاره نمود. یکی دیگر از نظریه‌هایی که در دهه‌های اخیر به تبیین علت زیر بنایی لکنت می پردازد تئوری یکپارچگی حسی حرکتی است. بر اساس این نظریه علت زیر بنایی لکنت مشکل در یکپارچگی حسی حرکتی[3] تولید گفتار می‌باشد [۱۰, ۱۱]. مدلی که براساس این نظریه در مورد لکنت مطرح شده است مدل دیوا[4] (DIVA) نام دارد. با توجه به تبیین دقیقی که مدل دیوا در مورد چگونگی به وجود آمدن لکنت ارائه می دهد در ادامه شرح مختصری بر اجزای این مدل و علت ایجاد لکنت براساس این مدل ارائه می‌دهیم. در مدل DIVA تعاملات حسی-حرکتی درگیر در کنترل اندام‌های گویایی در حین تولید گفتار توضیح داده می‌شود. در آخرین نسخه این مدل (۲۰۱۰) که لکنت بر اساس آن تبیین شده است سه زیر سیستم وجود دارد: ۱-زیرسیستم کنترل فیدبکی[5]، ۲- زیرسیستم کنترل فیدفورواردی[6]، و ۳- زیرسیستم نظارت[7] (پایش). در این مدل زیر سیستم کنترل فیدبکی به دو بخش تقسیم می شود ۱- زیرسیستم کنترل فیدبک شنیداری ۲- زیر سیستم کنترل فیدبک سوماتوسنسوری [۱۲]. به طور کلی در مدل دیوا تولید یک واج یا هجا با فعالیت سلولهای speech sound map آغاز می‌شود، که فرض می‌شود در کورتکس پیش حرکتی شکمی[8] قرار دارند. ناحیه‌ای که گاهی Frontal operculum هم نامیده می‌شود. پس از فعال شدن speech sound map، دستورات حرکتی از طریق دو زیر سیستم کنترلی فیدبکی و فیدفورواردی به کورتکس حرکتی می‌رسند. در سیستم کنترل فیدبکی سیگنالها از کورتکس پیش حرکتی به کورتکس حسی حرکتی و شنیداری منتقل می‌شوند و دستورات حسی برای صدا را رمزگذاری می‌نمایند. پروجکشن سیناپسی دیگری نیز از speech sound map به کورتکس حرکتی مدل می‌روند و دستورات حرکتی فیدفوروارد را می‌دهند. طی تولید صدا، نواحی حرکتی هدف با وضعیت شنیداری حال حاضر مقایسه می‌شود و هرگونه ناهمخوانی بین هدف و شکل وضعیت موجود، که خطای شنیداری[9] نامیده می‌شود، منجر به صدور فرمان به کورتکس حرکتی برای تصحیح این ناهمخوانی (از طریق پروجکشن از مناطق کورتکس شنیداری به کورتکس حرکتی) می‌شود. در اولین تلاش برای تولید، مدل دستورات فیدفورواردی تنظیم شده‌ای برای صدا ندارد، بنابراین ممکن است بر زیرسیستم کنترل فیدبک شنیداری تاکید بسیار داشته باشد. در هر تلاش برای تولید صدا، دستورات فیدفورواردی به روزرسانی می‌شود تا دستوراتی که توسط زیر سیستم کنترل فیدبک شنیداری در آن تلاش ایجاد می‌شود را ترکیب کند و این کار منجر به دستورات فیدفورداردی صحیح‌تری می‌شود. سرانجام دستور فیدفورواردی خودش برای تولید صدا در وضعیت طبیعی، کافی است. دستور فیدفورواردی به اندازه کافی دقیق است و درنتیجه تعداد بسیار کمی خطای شنیداری طی تولید صدا ایجاد می‌نماید و بنابراین دیگر زیرسیستم کنترل فیدبکی را به کمک فرا نمی‌خواند. در این مرحله مدل می‌تواند صداهای گفتاری را به صورت روان تولید کند [۱۳]. فرضیه مدل دیوا در مورد لکنت این است که افراد مبتلا به لکنت نسبت به افراد غیرلکنتی تکیه بیش از اندازه بر فیدبک حسی دارند.

---

[1] World Health Organization
[2] Heterogeneous
[3] Sensorimotor integration
[4] Directions into velocities of articulators
[5] Feedback control subsystem
[6] Feedforward control subsystem
[7] Monitoring subsystem
[8] Ventral premotor cortex
[9] Auditory Error

۲-۴-۵: تعریف متغیرها

متغیرهای پژوهش در دو گروه مستقل و وابسته قابل تقسیم هستند.

-تحریک tDCS (متغیر مستقل ،کیفی اسمی):

تعریف نظری: tDCS یک ابزار غیرتهاجمی است که از جریان الکتریکی مستقیم ضعیفی که از طریق جمجمه به بافت عصبی می‌رود، برای برانگیختی مغز استفاده می‌کند. این ابزار با اثر بر قطبیت غشاء نورون سبب تعدیل فعالیت عصبی خودبخودی مغز می‌گردد. بدین منظور، این دستگاه سه نوع تحریک الکتریکی متفاوت شامل: ۱) آند، ۲) کاتد، و ۳) شم ایجاد می‌نماید [۳۴].

تعریف کاربردی: دستگاه tDCS در گروه مداخله این مطالعه بر روی تحریک آند با شدت ۱ میلی آمپر تنظیم خواهد گردید که به عنوان تحریک فعال جهت ایجاد تغییر در عملکرد عصبی منطقه جایروس تمپورال فوقانی و منطقه خلفی میانی شنیداری است. در گروه مقایسه از حالت تحریک شم دستگاه با همان شدت جهت ایجاد شرایط مقایسه استفاده خواهد شد.

-تاخیر در فیدبک شنیداری (DAF)

تعریف نظری: در افراد طبیعی، صدای گفتار با تأخیری در حدود ۰/۰۰۱ ثانیه به گوش داخلی برمی‌گردد. تأخیر در فیدبک شنیداری شامل فاصله زمانی بین تولید صدای گفتاری و دریافت شنیداری آن است [۳۶].

تعریف کاربردی: در این مطالعه در هر دو گروه مداخله و مقایسه، شش جلسه درمانی برگزار خواهد شد که در هر جلسه، درمان با استفاده DAF انجام خواهد شد. مدت زمان تأخیر اعمال شده در این مطالعه ۲۰۰ میلی ثانیه خواهد بود [۳۷]. در گروه مداخله در ترکیب با تحریک tDCS واقعی و در گروه مقایسه در ترکیب با tDCS شم.

-ترکیب این دو عامل مستقل به صورت متغیری دو حالته شامل تحریک tDCS واقعی، همزمان با DAF و tDCS شم همزمان با DAF در تحلیل‌ها بکار خواهد رفت.

-نمره پرسشنامه SSI-4 (متغیر وابسته، کمی گسسته):

تعریف نظری: ابزار SSI4 ابزاری جهت تعیین شدت لکنت می‌باشد. این پرسشنامه رفتارهای قابل مشاهده لکنت شامل بسامد[27]، دیرش[28] و رفتارهای فیزیکی[29] همراه را بررسی می‌کند. روایی و پایایی این ابزار در سال ۲۰۱۲ در افراد لکنتی بزرگسال فارسی زبان مورد بررسی قرار گرفت و نویسندگان اعلام نمودند نسخه فارسی آزمون برای ارزیابی شدت لکنت در نمونه‌های فارسی زبان مناسب و بسیار کارآمد است [۳۸].

تعریف کاربردی: در مطالعه حاضر جهت تعیین شدت لکنت در ارزیابی‌های پیش از درمان، یک هفته و ۶ هفته پس از درمان از ابزار SSI-4 استفاده خواهد شد. نمره زیرمجموعه‌های آزمون و نیز نمره کلی آزمون به عنوان متغیرهای وابسته ثبت خواهند شد. نمره کل این آزمون از مجموع سه زیرمجموعه آزمون شامل بسامد، دیرش و رفتارهای فیزیکی همراه به دست می‌آید که حداقل این نمره ۸ و حداکثر آن ۵۶ می‌باشد.

-درصد هجاهای لکنت شده[30] SS% (متغیر وابسته، کمی پیوسته):

تعریف نظری: SS% مقیاسی سنتی برای اندازه‌گیری شدت لکنت می‌باشد که به این ترتیب محاسبه می‌شود: تعداد هجاهای لکنت شده در نمونه گفتار تقسیم بر کل هجاهای گفته شده در آن نمونه گفتار ضرب در صد [۳۹].

تعریف کاربردی: در این مطالعه مقدار SS% برای تکالیف خواندن با صدای بلند، روایت‌گویی و مکالمه قبل از درمان و نیز یک هفته و ۶ هفته بعد از درمان محاسبه خواهد شد. برای انجام تکلیف خواندن از متون ۱۰۰ کلمه‌ای پیش از درمان، یک هفته پس از درمان و ۶ هفته پس از درمان استفاده خواهد شد.

-امتیاز پرسشنامه ارزیابی جامع تجربه فرد از لکنت[31] (OASES) (متغیر وابسته، کمی گسسته):

---

27 Frequency

28 Duration

29 Physical concomitants

30 Percentage of stuttered syllables

31 Overall Assessment of the Speaker's Experience of Stuttering

افزایش برای تعداد جلسه DAF

۵) میانگین میزان اثر کلی و شدت اثر بخش‌های اطلاعات کلی، واکنش به لکنت، برقراری ارتباط در موقعیتهای روزمره، کیفیت زندگی و میزان اثر و شدت اثر کلی پرسشنامه OASES گروه مقایسه پیش از درمان با میانگین این نمرات یک هفته و ۶ هفته پس از درمان دارای تفاوت آماری معنادار است. با گروه مقایسه

۶) میانگین میزان اثر کلی و شدت اثر بخش‌های اطلاعات کلی، واکنش به لکنت، برقراری ارتباط در موقعیتهای روزمره، کیفیت زندگی و میزان اثر و شدت اثر کلی پرسشنامه OASES گروه مداخله پیش از درمان با میانگین این نمرات یک هفته و ۶ هفته پس از درمان دارای تفاوت آماری معنادار است.

۷) دامنه و تأخیر کامپوننت P300 گروه مداخله و گروه مقایسه پیش از درمان و یک هفته پس از درمان دارای تفاوت آماری معنادار است.

پیشنهاد دستیابی به ماندگاری متغیرها.

۸-۵. اهداف کاربردی

۱) بررسی سودمندی استفاده از دستگاه tDCS به عنوان درمان مکمل جهت افزایش روانی گفتار در افراد لکنتی بزرگسال

۲) بررسی ماندگاری اثرات افزایش روانی گفتار ایجاد شده با استفاده از tDCS و DAF در افراد لکنتی در طولانی مدت

۹-۵. جدول متغیرها

ردیف	عنوان متغیر	متغیر از نظر نقش آنها در تحقیق				متغیرها از نظر نوع				واحد اندازه گیری	نحوه اندازه‌گیری
		مستقل	وابسته	مداخله گر	زمینه‌ای	کمی		کیفی			
						پیوسته	گسسته	رتبه ای	اسمی		
۱	تحریک مغز	*							*		دستگاه tDCS و DAF
۲	نمره زیرآزمون بسامد SSI-4		*			*					محاسبه نمره زیرآزمون بسامد SSI-4-نمره بین۱۸-۲
۳	نمره زیرآزمون دیرش SSI-4		*			*					محاسبه نمره زیرآزمون دیرش SSI-4-نمره بین۱۸-۴
۴	نمره زیرآزمون رفتارهای فیزیکی همراه SSI-4		*				*				محاسبه نمره زیرآزمون رفتارهای فیزیکی همراه SSI-4-نمره بین۲۰-۰
۵	[illegible] SSI-4		*				*			–	محاسبه نمره آزمون SSI-4نمره بین ۵۶-۶
۶	میزان اثر کلی OASES		*			*				–	محاسبه میزان اثر کلی OASES - نمره بین ۲۰-۰
۷	شدت اثر کلی OASES		*					*		–	محاسبه شدت اثر کلی OASES - نمره بین ۲۰-۰

در نتیجه آسیب به سیستم کنترل فیدفورواردی افراد مبتلا به لکنت بیشتر بر استراتژی کنترل حرکتی بر پایه فیدبک تکیه می‌کنند. چنین آسیبی در سیستم کنترل فیدفورواردی قابل توجیه با این نظریه است که علت لکنت مهارت حرکتی کاهش یافته است. آسیب فرضی در کنترل فیدفورواردی و در نتیجه تکیه بیش از اندازه بر کنترل فیدبکی، خطاهای بیانی را افزایش می‌دهد. دستورات فیدفورواردی که دستورالعمل چگونگی عملکرد تولیدگرها را ذخیره می‌کند، به طور مستقیم از حافظه باز خوانی می‌شوند که در مدل دیوا از طریق پروجکشن از کورتکس پیش حرکتی به کورتکس حرکتی رخ می‌دهد. از طرف دیگر در کنترل فیدبکی باید خطاهای تولیدی (بیانی) کشف و تصحیح شود. از آنجایی که شناسایی و تصحیح خطاها بر پایه کنترل فیدبکی نسبتا کند است، تکیه بیش از اندازه بر کنترل فیدبکی منجر به تجمع خطا و سرانجام منجر به وقفه حرکتی[1] می‌شود و چنین وقفه ای ممکن است ایجاد تکرار هجا/صدا نماید [۱۴].

براساس تئوری یکپارچگی حسی حرکتی و مدل دیوا در مورد علت لکنت، درمان‌هایی نیز برای رفع لکنت و افزایش روانی گفتار مطرح شده‌اند. یکی از این درمان‌ها که بسیار مورد استفاده گفتاردرمانگران فعال در حوزه لکنت بوده است استفاده از تأخیر در فیدبک شنیداری[2] جهت اصلاح این سیستم و افزایش روانی گفتار می‌باشد [۱۵]. روش فیدبک شنیداری تأخیری به دلیل اینکه منجر به افزایش روانی گفتار و از بین رفتن لکنت می‌شود بسیار مورد توجه درمانگران بوده است، با این حال مشکلاتی در استفاده از این شیوه وجود دارد که به عنوان مثال می‌توان به طول دوره درمان طولانی جهت تثبیت روانی و کم بودن مدت ماندگاری اثر درمان اشاره نمود [۱۶].

از سوی دیگر، با توجه به دیدگاهای دیگری که نقص در سیستم‌های مغزی مربوط به تولید گفتار را در افراد مبتلا به لکنت تأیید کرده‌اند، در طول سال‌های اخیر روش‌های درمانی مکملی پیشنهاد کرده‌اند که نقایص پایه‌ای و زیربنایی مغزی را در افراد مبتلا به لکنت مورد هدف قرار می‌دهند تا درمان مؤثرتر و ماندگارتری ارائه شود. از جمله این روش‌ها استفاده از ابزارهای الکتروفیزیولوژیک است. یکی از روش‌های الکتروفیزیولوژیک که با هدف درمان اختلالات مغزی طراحی شده و مورد استفاده قرار گرفته‌اند، تحریک جریان مستقیم درون جمجمه‌ای[3] (tDCS) است [۱۷].

از نظر فیزیولوژیک می‌توان مکانیسم اثر tDCS را به این صورت تشریح نمود که tDCS شامل عبور یک جریان الکتریکی ضعیف ۱ یا ۲ میلی آمپری، بین دو الکترودی است که روی سر قرار می‌گیرند. مقداری از جریان (حدود ۱۰٪) از طریق جمجمه و قشر زیرین آن عبور می‌کند و برانگیختگی عصبی را با تغییر پتانسیل استراحت غشای نرونهای تحریک شده، تغییر می‌دهد [۱۸]. هر دستگاه tDCS شامل دو الکترود است، یک الکترود منفی که آند است و یک الکترود مثبت که کاتد می‌باشد. بر این اساس tDCS می‌تواند سه نوع محرک ایجاد کند: ۱) محرک آندال[4] که تحریک پذیری قشر را افزایش می‌دهد، ۲) محرک کاتدال[5] که عملکرد قشر را کاهش می‌دهد، و ۳) محرک شم[6] که یک جریان بسیار کوتاه مدت ایجاد می‌کند که مانند پلاسبو[7] عمل می‌کند و هیچ اثری بر عملکرد قشر نمی‌گذارد [۱۹]. معمولا پروتکل‌های tDCS از دیرش محرک به مدت بین ۱۰ تا ۲۰ دقیقه (همزمان با یک تکلیف) و سایز الکترود ۲۵cm^2 تا ۳۵cm^2 استفاده می‌کنند[۲۰]. اکثر مطالعات tDCS از جایگذاری الکترودی یک نیمکره‌ای استفاده کرده‌اند به این ترتیب که یک الکترود در ناحیه مورد نظر قرار داده می‌شود و الکترود دوم در ناحیه ای گذاشته می‌شود که از لحاظ عملکردی سالم است. هدف چنین جایگذاری تمرکز بر اثرات محرک در یک منطقه قشر است. گاهی اوقات وقتی که نیاز است تحریک یا مهار در دو نیمکره انجام شود از جایگذاری دو نیمکره‌ای استفاده می‌شود که الکترود دوم در ناحیه هومولوگ در نیمکره مقابل قرار می‌گیرد [۲۱]. به دلیل سهولت استفاده و کم بودن عوارض جانبی tDCS به

---

[1] Motor rest
[2] Delayed Auditory Feedback
[3] Transcranial Direct Current Stimulation
[4] Anodal
[5] Cathodal
[6] Sham
[7] Plasebo

ابزاری محبوب برای ایجاد تغییر در فعالیت مغزی به صورت غیرتهاجمی تبدیل شده است. پس از پایان درمان با tDCS می‌توان آزمونهای عملکردی یا پرسشنامه‌های سنجش مهارت و یا تکالیف مناسب را اجرا نمود تا از تاثیر درمانی tDCS بر عملکردهای بیمار اطمینان حاصل کرد، اما بهترین راه جهت نشان دادن تغییرات مغزی پس از استفاده از tDCS، استفاده از پتانسیل وابسته به رخداد[1] یا ERP می‌باشد. ERP تنها ابزاری است که می تواند وضوح زمانی میلی ثانیه ای را داشته باشد و پاسخ های فوری قشری به tDCS را اندازه گیری می کند. این ابزار، اجازه مطالعه غیر تهاجمی ارتباط کارکردی مناطق مختلف مغزی را می‌دهد [۲۲]. شواهدی وجود دارد که جریان الکتریکی ناشی از tDCS باعث افزایش برانگیختگی در نواحی زیر الکترود آند و مهار برانگیختگی در نواحی زیر الکترود کاتد می‌شود [۲۳]. با این وجود این مسأله که سایر متغیرها مانند جایگذاری الکترودها، شدت جریان و قطبیت تحریک چگونه بر این اثرات تأثیر می‌گذارند هنوز کاملا واضح نیست. استفاده از ERP به دلیل رزولوشن زمانی بسیار بالا کمک می‌کند مکانیسم‌های زیربنایی اثرات تحریک tDCS بیشتر مشخص شود [۲۴]. در شکل موج به دست آمده از ارزیابی با کمکERP، کامپوننت‌های مختلفی قابل بررسی هستند، یکی از کامپوننت‌هایی که در مطالعات در زمینه لکنت مورد بررسی قرار گرفته است P300 است. این کامپوننت ۳۰۰ میلی ثانیه بعد از درک محرک توسط فرد، در شکل موج قابل مشاهده است و مطالعات نشان داده‌اند که با استفاده از بررسی تغییرات در دامنه و تأخیر P300 می‌توان اثربخشی درمان را در افراد مبتلا به لکنت نشان داد؛ به این صورت که دامنه و تأخیر P300 در افراد مبتلا به لکنت پس از افزایش روانی گفتار به دامنه و تأخیر این کامپوننت در افراد طبیعی شبیه می‌شود[۲۵]

مطالعات اولیه با استفاده ازtDCS شامل تحریک کورتکس حرکتی اولیه بوده است. مطالعات زیادی با استفاده از tDCS در افراد دچار مشکلات گفتار و زبان اکتسابی انجام شده است [۲۶-۲۸]. در یک مقاله مروری سیستماتیک که مطالعات مربوط به آفازی را بررسی نموده است، سودمندی این ابزار را در بهبود مهارتهای حرکتی و بیانی گفتار در این جمعیت نشان داده است[۲۹]. همچنین در دو مطالعه بهبود مهارت حرکتی گفتار در بیماران مبتلا به آپراکسی اکتسابی، با استفاده از tDCS در ناحیه جایروس فرونتال تحتانی چپ مشاهده شد. این مطالعات نقش تاثیرگذار tDCS در توانبخشی حرکتی گفتار را نشان داده‌اند[۳۰, ۳۱]. نتایج به دست آمده از این مطالعات، پژوهشگران را برای استفاده از tDCS به عنوان یک روش مکمل در درمان روانی گفتار در بزرگسالان مبتلا به لکنت ترغیب کرد و اخیرا در دو مطالعه از tDCS به عنوان درمان مکمل و تسهیل کننده جهت افزایش روانی گفتار و ثبات نتایج درمان استفاده شده است [۲۱, ۳۲].

چنانچه در بالا ذکر شد مطالعات محدودی اخیرا به بررسی تاثیر tDCS در درمان لکنت پرداخته‌اند. در دو مطالعه‌ای که تا کنون با استفاده از tDCS بر روی افراد مبتلا به لکنت انجام شده‌است تحریک در ناحیه فرونتال ارائه شده است. در حال حاضر این سؤال مطرح است که آیا با توجه به نظریه‌های دیگر در مورد آسیب سایر نواحی مغزی در لکنت (نظریه یکپارچگی حسی- حرکتی)، تحریک نواحی دیگر نیز می‌تواند باعث افزایش روانی شود یا خیر. از سوی دیگر تکالیف مورد استفاده در این مطالعات گفتار همزمان[2] و خواندن همزمان[3] بوده است در نتیجه اثر استفاده از tDCS همزمان با سایر روش‌های افزایش روانی گفتار همچنان مورد سوال است. با توجه به اینکه این مطالعه بر اساس فرضیه نقص در فیدبک شنیداری استوار است، بنابراین جهت افزایش روانی گفتار از روش فیدبک شنیداری تأخیری استفاده خواهیم کرد و به دلیل اینکه روانی گفتار ایجاد شده در روش تأخیر در فیدبک شنیداری موقت است و با حذف تاخیر، ناروانی تا حدودی به حالت اول برمی‌گردد، جهت حفظ طولانی مدت تغییرات از روش مکمل tDCS استفاده خواهد شد. بنابراین هدف این مطالعه بررسی تاثیر رویکرد درمانی ترکیبی فیدبک شنیداری تاخیری و تحریک جریان مستقیم درون جمجمه‌ای برای افزایش روانی گفتار در بزرگسالان مبتلا به لکنت است. در این مطالعه که بر روی ۵۰ بزرگسال مبتلا به لکنت (۲۵ نفر گروه مداخله و ۲۵ نفر گروه مقایسه) انجام می‌شود با کمک تحریک الکتریکی

[1] Event Related Potential

[2] Choral speech

[3] Choral reading

۲-۴-۵: تعریف متغیرها

متغیرهای پژوهش در دو گروه مستقل و وابسته قابل تقسیم هستند.

-تحریک tDCS (متغیر مستقل ،کیفی اسمی):

تعریف نظری: tDCS یک ابزار غیرتهاجمی است که از جریان الکتریکی مستقیم ضعیفی که از طریق جمجمه به بافت عصبی می‌رود، برای برانگیختی مغز استفاده می‌کند. این ابزار با اثر بر قطبیت غشاء نورون سبب تعدیل فعالیت عصبی خودبخودی مغز می‌گردد. بدین منظور، این دستگاه سه نوع تحریک الکتریکی متفاوت شامل: ۱) آند، ۲) کاتد، و ۳) شم ایجاد می‌نماید [۳۴].

تعریف کاربردی: دستگاه tDCS در گروه مداخله این مطالعه بر روی تحریک آند با شدت ۱ میلی آمپر تنظیم خواهد گردید که به عنوان تحریک فعال جهت ایجاد تغییر در عملکرد عصبی منطقه جایروس تمپورال فوقانی و منطقه خلفی میانی شنیداری است. در گروه مقایسه از حالت تحریک شم دستگاه با همان شدت جهت ایجاد شرایط مقایسه استفاده خواهد شد.

-تأخیر در فیدبک شنیداری (DAF)

تعریف نظری: در افراد طبیعی، صدای گفتار با تأخیری در حدود ۰/۰۰۱ ثانیه به گوش داخلی برمی‌گردد. تأخیر در فیدبک شنیداری شامل فاصله زمانی بین تولید صدای گفتاری و دریافت شنیداری آن است [۳۶].

تعریف کاربردی: در این مطالعه در هر دو گروه مداخله و مقایسه، شش جلسه درمانی برگزار خواهد شد که در هر جلسه، درمان با استفاده DAF انجام خواهد شد. مدت زمان تأخیر اعمال شده در این مطالعه ۲۰۰ میلی ثانیه خواهد بود [۳۷]. در گروه مداخله در ترکیب با تحریک tDCS واقعی و در گروه مقایسه در ترکیب با tDCS شم.

-ترکیب این دو عامل مستقل به صورت متغیری دو حالته شامل تحریک tDCS واقعی، همزمان با DAF و tDCS شم همزمان با DAF در تحلیل‌ها بکار خواهد رفت.

-نمره پرسشنامه SSI-4 (متغیر وابسته، کمی گسسته):

تعریف نظری: ابزار SSI4 ابزاری جهت تعیین شدت لکنت می‌باشد. این پرسشنامه رفتارهای قابل مشاهده لکنت شامل بسامد[1]، دیرش[2] و رفتارهای فیزیکی[3] همراه را بررسی می‌کند. روایی و پایایی این ابزار در سال ۲۰۱۲ در افراد لکنتی بزرگسال فارسی زبان مورد بررسی قرار گرفت و نویسندگان اعلام نمودند نسخه فارسی آزمون برای ارزیابی شدت لکنت در نمونه‌های فارسی زبان مناسب و بسیار کارآمد است [۳۸].

تعریف کاربردی: در مطالعه حاضر جهت تعیین شدت لکنت در ارزیابی‌های پیش از درمان، یک هفته و ۶ هفته پس از درمان از ابزار SSI-4 استفاده خواهد شد. نمره زیرمجموعه‌های آزمون و نیز نمره کلی آزمون به عنوان متغیرهای وابسته ثبت خواهند شد. نمره کل این آزمون از مجموع سه زیرمجموعه آزمون شامل بسامد، دیرش و رفتارهای فیزیکی همراه به دست می‌آید که حداقل این نمره ۸ و حداکثر آن ۵۶ می‌باشد.

-درصد هجاهای لکنت شده[4] SS% (متغیر وابسته، کمی پیوسته):

تعریف نظری: SS% مقیاسی سنتی برای اندازه‌گیری شدت لکنت می‌باشد که به این ترتیب محاسبه می‌شود: تعداد هجاهای لکنت شده در نمونه گفتار تقسیم بر کل هجاهای گفته شده در آن نمونه گفتار ضرب در صد [۳۹].

تعریف کاربردی: در این مطالعه مقدار SS% برای تکالیف خواندن با صدای بلند، روایت‌گویی و مکالمه قبل از درمان و نیز یک هفته و ۶ هفته بعد از درمان محاسبه خواهد شد. برای انجام تکلیف خواندن از متون ۱۰۰ کلمه‌ای پیش از درمان، یک هفته پس از درمان و ۶ هفته پس از درمان استفاده خواهد شد.

-امتیاز پرسشنامه ارزیابی جامع تجربه فرد از لکنت[5] (OASES) (متغیر وابسته، کمی گسسته):

---

1 Frequency

2 Duration

3 Physical concomitants

4 Percentage of stuttered syllables

5 Overall Assessment of the Speaker's Experience of Stuttering

الکتریکی ضعیفی که در نواحی جایروس گیجگاهی فوقانی[۲۰] و ناحیه خلفی-میانی شنیداری[۲۱] [۳۳] ارائه خواهد شد. تغییرات ایجاد شده در روانی گفتار و ماندگاری درمان بررسی خواهد شد. ارزیابی‌های قبل و بعد از درمان شامل ارزیابی های رفتاری و الکتروفیزیولوژکال (ERP) خواهد بود.

۵-۲. جنبه جدید بودن موضوع

به طور کلی استفاده از tDCS در مشکلات گفتار و زبان مبحث جدیدی است و در دهه‌های اخیر با مطالعات در جمعیت افراد مبتلا به آفازی و آپراکسی آغاز شده است. در همین راستا، سابقه مطالعه با استفاده از درمان مکمل tDCS در افراد مبتلا به لکنت به حدود سه سال اخیر برمی‌گردد، به طوری که اولین مطالعات در سال ۲۰۱۷ انجام شده‌اند [۳۲]. با توجه به جدید بودن مطالعه با استفاده از tDCS، تاکنون مطالعاتی که در جهان بر روی لکنت و با استفاده از این ابزار انجام شده‌اند از روش گفتار همزمان و یا خواندن همزمان استفاده نموده‌اند، در حالی که در این مطالعه برای اولین بار روش ترکیبی استفاده از فیدبک شنیداری تأخیری و تحریک tDCS استفاده خواهد شد. از سوی دیگر، مطالعات پیشین از تحریک tDCS در نواحی فرونتال استفاده کرده‌اند، در حالی که در مطالعه حاضر با توجه به نظریهٔ زیربنایی مطالعه، از تحریک نواحی تمپورال استفاده خواهد شد و این یکی دیگر از موارد جدید بودن این مطالعه است.

۵-۳. سابقه علمی پژوهش‌های انجام شده

۵-۳-۱. پژوهش‌های خارجی

در مطالعه‌ای توماسن[۲۲] در سال ۲۰۱۷ در یک مطالعه تک نمونه‌ای[۲۳] و تک جلسه‌ای که بر روی یک فرد بزرگسال مبتلا به لکنت و یک فرد طبیعی انجام شد، اثر استفاده از tDCS در ناحیه پیش حرکتی[۲۴] را بر افزایش روانی گفتار در حین تکلیف خواندن همزمان را مورد بررسی قرار داد. وی در این مطالعه الکترود کاتد (الکترود مهاری) را بر روی کورتکس پیش حرکتی چپ و الکترود آند (الکترود تحریکی) را بر روی ناحیه مکمل حرکتی[۲۵] راست قرار داد و روانی گفتار را در زمان اجرای خواندن همزمان قبل، حین و بعد از استفاده از tDCS بررسی نمود. در این مطالعه چهار حالت مورد بررسی قرار گرفت؛ در دو حالت فرد مبتلا به لکنت و فرد طبیعی هر دو tDCS واقعی را دریافت کردند و تکلیف خواندن همزمان را اجرا کردند، در دو حالت دیگر فرد مبتلا به لکنت و فرد طبیعی هر دو tDCS شم را دریافت کردند و مجدداً تکلیف خواندن همزمان را انجام دادند. وی گزارش کرد در فرد مبتلا به لکنت در حین انجام تکلیف هم در حضور محرک واقعی و هم تحت محرک شم لکنت تشدید شد و با قطع محرک tDCS لکنت فرد به حالت پایه برمی‌گشت و در فرد طبیعی در هیچ حالتی لکنت مشاهده نشد [۳۲]. مطالعه توماسن به صورت تک نمونه‌ای انجام شد و به همین دلیل نمی‌توان در مورد تعمیم مطالعه وی به جمعیت افراد مبتلا به لکنت با اطمینان بحث کرد و به نظر می‌رسد بهتر است این مطالعه در حجم نمونه بیشتری انجام شود و نتایج مورد بررسی قرار گیرد.

چستر[۲۶] در مطالعه‌ای که در سال ۲۰۱۷ انجام داد به بررسی این موضوع پرداخت که آیا یک جلسه tDCS می‌تواند روانی گفتار را در افراد مبتلا به لکنت بهبود دهد. ۱۶ فرد بزرگسال مبتلا به لکنت رشدی با میانگین سنی ۳۰ سال در مطالعه وی شرکت کردند. در یک جلسه

---

[20] Superior temporal gyrus

[21] Posterior-medial auditory area

[22] Thomason

[23] Single-subject

[24] Premotor

[25] Supplementary motor area

[26] Chester

درمان های ترکیبی در زمینه وجود ندارد؟
اگر موجود است تفاوت ها ذکر گشته است.

چرا رویکرد درمانی؟

- بر میزان بهبودی روانی گفتار
- بر میزان عملکرد روانی گفتار

شماره:

تاریخ:

پیوست:

فرم مخصوص ثبت پایان نامه کارشناسی ارشد و دکتری

۱. اطلاعات مربوط به دانشجو

نام و نام خانوادگی: نرگس معین	شماره دانشجویی: ۹۴۲۳۳۶۳۰۰۲
رشته تحصیلی: گفتاردرمانی	مقطع تحصیلی: دکترای تخصصی
آدرس: تهران، بلوار میرداماد، میدان مادر، خیابان شاه‌نظری، خیابان مددکاران، دانشکده علوم توانبخشی دانشگاه علوم پزشکی ایران، گروه گفتاردرمانی	تلفن: ۲۲۲۲۸۰۵۲
آدرس پست الکترونیکی: moin.narges@gmail.com	تلفن همراه: ۰۹۱۶۳۹۸۱۸۳۵

۲. عنوان پایان‌نامه/رساله

فارسی: تأثیر رویکرد درمانی ترکیبی فیدبک شنیداری تاخیری و تحریک جریان مستقیم درون جمجمه‌ای برای افزایش روانی گفتار در بزرگسالان مبتلا به لکنت

English: The effect of the combined treatment approach of Delayed Auditory Feedback and trans-cranial Direct Current Stimulation to enhancement of speech fluency in adults who stutter

واژه های کلیدی

فارسی: لکنت، روانی گفتار، فیدبک شنیداری تأخیری، تحریک جریان مستقیم درون جمجمه ای

S F Trans Cranial

English: stuttering, Speech fluency, Delayed Auditory Feedback, trans-cranial Direct Current Stimulation

۳. مشخصات استاد راهنمای اول

نام‌خانوادگی: محمدی نام: ریحانه	تخصص اصلی: گفتاردرمانی
آخرین مدرک تحصیلی و رتبه دانشگاهی: دکترای تخصصی گفتاردرمانی-استادیار	ایمیل: mohamadi.re88@gmail.com
آدرس: تهران، بلوار میرداماد، میدان مادر، خیابان شاه‌نظری، خیابان مددکاران، دانشکده علوم توانبخشی دانشگاه علوم پزشکی ایران، گروه گفتاردرمانی	تلفن: ۲۲۲۲۸۰۵۲

۳. مشخصات استاد راهنمای دوم

۴) مقایسه میانگین نمره زیرآزمون‌های بسامد هجاهای لکنت شده، دیرش لکنت، رفتارهای فیزیکی همراه و نمره کلی پرسشنامه SSI-4 گروه مقایسه پیش از درمان با میانگین نمرات این زیرآزمون یک هفته و ۶ هفته پس از درمان.

۵) مقایسه میانگین میزان اثر کلی و شدت اثر بخش‌های اطلاعات کلی، واکنش به لکنت، برقراری ارتباط در موقعیتهای روزمره، کیفیت زندگی و میزان اثر و شدت اثر کلی پرسشنامه OASES گروه مقایسه پیش از درمان با میانگین این نمرات یک هفته و ۶ هفته پس از درمان.

۶) مقایسه میانگین میزان اثر کلی و شدت اثر بخش‌های اطلاعات کلی، واکنش به لکنت، برقراری ارتباط در موقعیتهای روزمره، کیفیت زندگی و میزان اثر و شدت اثر کلی پرسشنامه OASES گروه مداخله پیش از درمان با میانگین این نمرات یک هفته و ۶ هفته پس از درمان.

مقایسه دو گروه مداخله و کنترل باید آورده شود؟

۷) مقایسه دامنه و تأخیر کامپوننت P300 گروه مداخله و گروه مقایسه پیش از درمان و یک هفته پس از درمان.

۵-۷. سوالات و یا فرضیات پژوهش

۵-۷-۱. سوالات پژوهش

۱) میانگین درصد هجای لکنت شده (SS%) در گروه مداخله و گروه مقایسه به طور مجزا پیش از درمان، یک هفته و ۶ هفته پس از درمان چقدر است؟

۲) میانگین نمره زیرآزمون‌های بسامد هجاهای لکنت شده، دیرش لکنت، رفتارهای فیزیکی همراه و نمره کلی پرسشنامه SSI-4 در گروه مداخله و گروه مقایسه به طور مجزا پیش از درمان، یک هفته و ۶ هفته پس از درمان چقدر است؟

۳) میانگین میزان اثر کلی و شدت اثر بخش‌های اطلاعات کلی، واکنش به لکنت، برقراری ارتباط در موقعیتهای روزمره، کیفیت زندگی و میزان اثر و شدت اثر کلی پرسشنامه OASES در گروه مداخله و گروه مقایسه به طور مجزا پیش از درمان، یک هفته و ۶ هفته پس از درمان چقدر است؟

۴) دامنه و تأخیر کامپوننت P300 در گروه مداخله و گروه مقایسه پیش از درمان و یک هفته پس از درمان چقدر است؟

۵-۷-۲. فرضیات پژوهش

۱) میانگین درصد هجای لکنت شده (SS%) در گروه مداخله پیش از درمان با میانگین این نمره یک هفته و ۶ هفته پس از درمان دارای تفاوت آماری معنادار است.

۲) میانگین درصد هجای لکنت شده (SS%) در گروه مقایسه پیش از درمان با میانگین این نمره یک هفته و ۶ هفته پس از درمان دارای تفاوت آماری معنادار است.

۳) میانگین نمره زیرآزمون‌های بسامد هجاهای لکنت شده، دیرش لکنت، رفتارهای فیزیکی همراه و نمره کلی پرسشنامه SSI-4 گروه مداخله پیش از درمان با میانگین این نمرات یک هفته و ۶ هفته پس از درمان دارای تفاوت آماری معنادار است.

۴) میانگین نمره زیرآزمون‌های بسامد هجاهای لکنت شده، دیرش لکنت، رفتارهای فیزیکی همراه و نمره کلی پرسشنامه SSI-4 گروه مقایسه پیش از درمان با میانگین نمرات این زیرآزمون یک هفته و ۶ هفته پس از درمان دارای تفاوت آماری معنادار است.

۵) میانگین میزان اثر کلی و شدت اثر بخش‌های اطلاعات کلی، واکنش به لکنت، برقراری ارتباط در موقعیتهای روزمره، کیفیت زندگی و میزان اثر و شدت اثر کلی پرسشنامه OASES گروه مقایسه پیش از درمان با میانگین این نمرات یک هفته و ۶ هفته پس از درمان دارای تفاوت آماری معنادار است.

۶) میانگین میزان اثر کلی و شدت اثر بخش‌های اطلاعات کلی، واکنش به لکنت، برقراری ارتباط در موقعیتهای روزمره، کیفیت زندگی و میزان اثر و شدت اثر کلی پرسشنامه OASES گروه مداخله پیش از درمان با میانگین این نمرات یک هفته و ۶ هفته پس از درمان دارای تفاوت آماری معنادار است.

آسیبی در سیستم کنترل فیدفورواردی قابل توجیه با این نظریه است که علت لکنت مهارت حرکتی کاهش یافته است. آسیب فرضی در کنترل فیدفورواردی و در نتیجه تکیه بیش از اندازه بر کنترل فیدبکی، خطاهای بیانی را افزایش می‌دهد. دستورات فیدفورواردی که دستورالعمل چگونگی عملکرد تولیدگرها را ذخیره می‌کند، به طور مستقیم از حافظه باز خوانی می‌شوند که در مدل دیوا از طریق پروجکشن از کورتکس پیش حرکتی به کورتکس حرکتی رخ می‌دهد. از طرف دیگر در کنترل فیدبکی باید خطاهای تولیدی (بیانی) کشف و تصحیح شود. از آنجایی که شناسایی و تصحیح خطاها بر پایه کنترل فیدبکی نسبتا کند است، تکیه بیش از اندازه بر کنترل فیدبکی منجر به تجمع خطا و سرانجام منجر به وقفه حرکتی[1] می‌شود و چنین وقفه ای ممکن است ایجاد تکرار هجا/صدا نماید [۱۴]. بهتر است مقاله مرتبط ارائه شود

براساس تئوری یکپارچگی حسی حرکتی و مدل دیوا در مورد علت لکنت، درمان‌هایی نیز برای رفع لکنت و افزایش روانی گفتار مطرح شده‌اند. یکی از این درمان‌ها که بسیار مورد استفاده گفتاردرمانگران فعال در حوزه لکنت بوده است استفاده از تـأخیر در فیـدبک شـنیداری[2] جهت اصلاح این سیستم و افزایش روانی گفتار می‌باشد [۱۵]. روش فیدبک شنیداری تأخیری به دلیل اینکه منجر به افزایش روانی گفتـار و از بـین رفتن لکنت می‌شود بسیار مورد توجه درمانگران بوده است، با این حال مشکلاتی در استفاده از این شیوه وجود دارد که به عنـوان مثـال مـی‌توان به طول دوره درمان طولانی جهت تثبیت روانی و کم بودن مدت ماندگاری اثر درمان اشاره نمود [۱۶].

از سوی دیگر، با توجه به دیدگاهای دیگری که نقص در سیستم‌های مغزی مربوط به تولید گفتار را در افراد مبتلا به لکنت تأیید کرده‌اند، در طول سال‌های اخیر روش‌های درمانی مکملی پیشنهاد کرده‌اند که نقایص پایه‌ای و زیربنایی مغزی را در افراد مبتلا به لکنت مورد هدف قرار می‌دهند تا درمان مؤثرتر و ماندگارتری ارائه شود. از جمله این روش‌ها استفاده از ابزارهای الکتروفیزیولوژیک است. یکی از روش‌های الکتروفیزیولوژیک که با هدف درمان اختلالات مغزی طراحی شده و مورد استفاده قرار گرفته‌اند، تحریک جریان مستقیم درون جمجمه‌ای[3] (tDCS) است [۱۷].

این مقاله فقط نظریه عصبی تولید و [illegible] بر اساس [illegible] اصطلاح [illegible] لکنت ؟

از نظر فیزیولوژیک می‌توان مکانیسم اثر tDCS را به این صورت تشریح نمود که tDCS شامل عبور یک جریان الکتریکی ضعیف ۱ یا ۲ میلی آمپری، بین دو الکترودی است که روی سر قرار می‌گیرند. مقداری از جریان (حدود ۱۰٪) از طریق جمجمه و قشر زیرین آن عبور می‌کند و برانگیختگی عصبی را با تغییر پتانسیل استراحت غشای نرونهای تحریک شده، تغییر می‌دهد [۱۸]. هر دستگاه tDCS شامل دو الکترود است، یک الکترود منفی که آند است و یک الکترود مثبت که کاتد می‌باشد. بر این اساس tDCS می‌تواند سه نوع محرک ایجاد کند: ۱) محرک آندال[4] که تحریک پذیری قشر را افزایش می‌دهد، ۲) محرک کاتدال[5] که عملکرد قشر را کاهش می‌دهد، و ۳) محرک شم[6] که یک جریان بسیار کوتاه مدت ایجاد می‌کند که مانند پلاسبو[7] عمل می‌کند و هیچ اثری بر عملکرد قشر نمی‌گذارد [۱۹]. معمولا پروتکل‌های tDCS از دیرش محرک به مدت بین ۱۰ تا ۲۰ دقیقه (همزمان با یک تکلیف) و سایز الکترود ۲۵cm^2 تا ۳۵cm^2 استفاده می‌کنند[۲۰]. اکثر مطالعات tDCS از جایگذاری الکترودی یک نیمکره‌ای استفاده کرده‌اند به این ترتیب که یک الکترود در ناحیه مورد نظر قرار داده می‌شود و الکترود دوم در ناحیه ای گذاشته می‌شود که از لحاظ عملکردی سالم است. هدف چنین جایگذاری تمرکز بر اثرات محرک در یک منطقه قشر است. گاهی اوقات وقتی که نیاز است تحریک یا مهار در دو نیمکره انجام شود از جایگذاری دو نیمکره‌ای استفاده می‌شود که الکترود دوم در ناحیه هومولوگ در نیمکره مقابل قرار می‌گیرد [۲۱]. به دلیل سهولت استفاده و کم بودن عوارض جانبی tDCS به ابزاری محبوب برای ایجاد تغییر در فعالیت مغزی به صورت غیرتهاجمی تبدیل شده است. پس از پایان درمان با tDCS می‌توان آزمونهای عملکردی یا پرسشنامه‌های سنجش مهارت و یا تکالیف مناسب را اجرا نمود تا از تاثیر درمانی tDCS بر عملکردهای بیمار اطمینان حاصل

[1] Motor rest
[2] Delayed Auditory Feedback
[3] Transcranial Direct Current Stimulation
[4] Anodal
[5] Cathodal
[6] Sham
[7] Plasebo

ابزاری محبوب برای ایجاد تغییر در فعالیت مغزی به صورت غیرتهاجمی تبدیل شده است. پس از پایان درمان با tDCS می‌توان آزمونهای عملکردی یا پرسشنامه‌های سنجش مهارت و یا تکالیف مناسب را اجرا نمود تا از تاثیر درمانی tDCS بر عملکردهای بیمار اطمینان حاصل کرد، اما بهترین راه جهت نشان دادن تغییرات مغزی پس از استفاده از tDCS، استفاده از پتانسیل وابسته به رخداد[17] یا ERP می‌باشد. ERP تنها ابزاری است که می تواند وضوح زمانی میلی ثانیه ای را داشته باشد و پاسخ های فوری قشری به tDCS را اندازه گیری می کند. این ابزار، اجازه مطالعه غیر تهاجمی ارتباط کارکردی مناطق مختلف مغزی را می‌دهد [۲۲]. شواهدی وجود دارد که جریان الکتریکی ناشی از tDCS باعث افزایش برانگیختگی در نواحی زیر الکترود آند و مهار برانگیختگی در نواحی زیر الکترود کاتد می‌شود [۲۳]. با این وجود این مسأله که سایر متغیرها مانند جایگذاری الکترودها، شدت جریان و قطبیت تحریک چگونه بر این اثرات تأثیر می‌گذارند هنوز کاملا واضح نیست. استفاده از ERP به دلیل رزولوشن زمانی بسیار بالا کمک می‌کند مکانیسم‌های زیربنایی اثرات تحریک tDCS بیشتر مشخص شود [۲۴]. در شکل موج به دست آمده از ارزیابی با کمکERP، کامپوننت‌های مختلفی قابل بررسی هستند، یکی از کامپوننت‌هایی که در مطالعات در زمینه لکنت مورد بررسی قرار گرفته است P300 است. این کامپوننت ۳۰۰ میلی ثانیه بعد از درک محرک توسط فرد، در شکل موج قابل مشاهده است و مطالعات نشان داده‌اند که با استفاده از بررسی تغییرات در دامنه و تأخیر P300 می‌توان اثربخشی درمان را در افراد مبتلا به لکنت نشان داد؛ به این صورت که دامنه و تأخیر P300 در افراد مبتلا به لکنت پس از افزایش روانی گفتار به دامنه و تأخیر این کامپوننت در افراد طبیعی شبیه می‌شود[۲۵]

مطالعات اولیه با استفاده ازtDCS شامل تحریک کورتکس حرکتی اولیه بوده است. مطالعات زیادی با استفاده از tDCS در افراد دچار مشکلات گفتار و زبان اکتسابی انجام شده است [۲۶-۲۸]. در یک مقاله مروری سیستماتیک که مطالعات مربوط به آفازی را بررسی نموده است، سودمندی این ابزار را در بهبود مهارتهای حرکتی و بیانی گفتار در این جمعیت نشان داده است[۲۹]. همچنین در دو مطالعه بهبود مهارت حرکتی گفتار در بیماران مبتلا به آپراکسی اکتسابی، با استفاده از tDCS در ناحیه جایروس فرونتال تحتانی چپ مشاهده شد. این مطالعات نقش تاثیرگذار tDCS در توانبخشی حرکتی گفتار را نشان داده‌اند[۳۰, ۳۱]. نتایج به دست آمده از این مطالعات، پژوهشگران را برای استفاده از tDCS به عنوان یک روش مکمل در درمان روانی گفتار در بزرگسالان مبتلا به لکنت ترغیب کرد و اخیرا در دو مطالعه از tDCS به عنوان درمان مکمل و تسهیل کننده جهت افزایش روانی گفتار و ثبات نتایج درمان استفاده شده است [۲۱, ۳۲].

چنانچه در بالا ذکر شد مطالعات محدودی اخیرا به بررسی تاثیر tDCS در درمان لکنت پرداخته‌اند. در دو مطالعه‌ای که تا کنون با استفاده از tDCS بر روی افراد مبتلا به لکنت انجام شده‌است تحریک در ناحیه فرونتال ارائه شده است. در حال حاضر این سؤال مطرح است که آیا با توجه به نظریه‌های دیگر در مورد آسیب سایر نواحی مغزی در لکنت (نظریه یکپارچگی حسی- حرکتی)، تحریک نواحی دیگر نیز می‌تواند باعث افزایش روانی شود یا خیر. از سوی دیگر تکالیف مورد استفاده در این مطالعات گفتار همزمان[18] و خواندن همزمان[19] بوده است در نتیجه اثر استفاده از tDCS همزمان با سایر روش‌های افزایش روانی گفتار همچنان مورد سوال است. با توجه به اینکه این مطالعه بر اساس فرضیه نقص در فیدبک شنیداری استوار است، بنابراین جهت افزایش روانی گفتار از روش فیدبک شنیداری تأخیری استفاده خواهیم کرد و به دلیل اینکه روانی گفتار ایجاد شده در روش تأخیر در فیدبک شنیداری موقت است و با حذف تاخیر، ناروانی تا حدودی به حالت اول برمی‌گردد، جهت حفظ طولانی مدت تغییرات از روش مکمل tDCS استفاده خواهد شد. بنابراین هدف این مطالعه بررسی تاثیر رویکرد درمانی ترکیبی فیدبک شنیداری تاخیری و تحریک جریان مستقیم درون جمجمه‌ای برای افزایش روانی گفتار در بزرگسالان مبتلا به لکنت است. در این مطالعه که بر روی ۵۰ بزرگسال مبتلا به لکنت (۲۵ نفر گروه مداخله و ۲۵ نفر گروه مقایسه) انجام می‌شود. با کمک تحریک

به نظر می‌رسد حجم اطلاعات در بخش مقدمه و بیان مسئله بسیار است
و ارجحیت این روش و مزایای آن در
مقایسه با روشهای سنتی روان‌سازی گفتار مثبت شده است
که به نظر می‌رسد جای این مبحث خالی است.

[17] Event Related Potential

[18] Choral speech

[19] Choral reading

جایگاه آن کامپوننت را در شکل موج نشان می‌دهد. یکی از کامپوننت‌های مهم در مطالعات P300 است که در ۳۰۰ میلی ثانیه پس از درک محرک غیرمنتظره رخ می‌دهد [۳۵].

۲-۴-۵: تعریف متغیرها

متغیرهای پژوهش در دو گروه مستقل و وابسته قابل تقسیم هستند.

-تحریک tDCS (متغیر مستقل ،کیفی اسمی):

تعریف نظری: tDCS یک ابزار غیرتهاجمی است که از جریان الکتریکی مستقیم ضعیفی که از طریق جمجمه به بافت عصبی می‌رود، برای برانگیختی مغز استفاده می‌کند. این ابزار با اثر بر قطبیت غشاء نورون سبب تعدیل فعالیت عصبی خودبخودی مغز می‌گردد. بدین منظور، این دستگاه سه نوع تحریک الکتریکی متفاوت شامل: ۱) آند، ۲) کاتد، و ۳) شم ایجاد می‌نماید [۳۴].

تعریف کاربردی: دستگاه tDCS در گروه مداخله این مطالعه بر روی تحریک آند با شدت ۱ میلی آمپر تنظیم خواهد گردید که به عنوان تحریک فعال جهت ایجاد تغییر در عملکرد عصبی منطقه جایروس تمپورال فوقانی و منطقه خلفی میانی شنیداری است. در گروه مقایسه از حالت تحریک شم دستگاه با همان شدت جهت ایجاد شرایط مقایسه استفاده خواهد شد.

-تأخیر در فیدبک شنیداری (DAF)

تعریف نظری: در افراد طبیعی، صدای گفتار با تأخیری در حدود ۰/۰۰۱ ثانیه به گوش داخلی برمی‌گردد. تأخیر در فیدبک شنیداری شامل فاصله زمانی بین تولید صدای گفتاری و دریافت شنیداری آن است [۳۶].

تعریف کاربردی: در این مطالعه در هر دو گروه مداخله و مقایسه، شش جلسه درمانی برگزار خواهد شد که در هر جلسه، درمان با استفاده DAF انجام خواهد شد. مدت زمان تأخیر اعمال شده در این مطالعه ۲۰۰ میلی ثانیه خواهد بود [۳۷]. در گروه مداخله در ترکیب با تحریک tDCS واقعی و در گروه مقایسه در ترکیب با tDCS شم.

-ترکیب این دو عامل مستقل به صورت متغیری دو حالته شامل تحریک tDCS واقعی، همزمان با DAF و tDCS شم همزمان با DAF در تحلیل‌ها بکار خواهد رفت.

-نمره پرسشنامه SSI-4 (متغیر وابسته، کمی گسسته):

تعریف نظری: ابزار SSI4 ابزاری جهت تعیین شدت لکنت می‌باشد. این پرسشنامه رفتارهای قابل مشاهده لکنت شامل بسامد[28]، دیرش[29] و رفتارهای فیزیکی[30] همراه را بررسی می‌کند. روایی و پایایی این ابزار در سال ۲۰۱۲ در افراد لکنتی بزرگسال فارسی زبان مورد بررسی قرار گرفت و نویسندگان اعلام نمودند نسخه فارسی آزمون برای ارزیابی شدت لکنت در نمونه‌های فارسی زبان مناسب و بسیار کارآمد است [۳۸].

تعریف کاربردی: در مطالعه حاضر جهت تعیین شدت لکنت در ارزیابی‌های پیش از درمان، یک هفته و ۶ هفته پس از درمان از ابزار SSI-4 استفاده خواهد شد. نمره زیرمجموعه‌های آزمون و نیز نمره کلی آزمون به عنوان متغیرهای وابسته ثبت خواهند شد. نمره کل این آزمون از مجموع سه زیرمجموعه آزمون شامل بسامد، دیرش و رفتارهای فیزیکی همراه به دست می‌آید که حداقل این نمره ۸ و حداکثر آن ۵۶ می‌باشد.

-درصد هجاهای لکنت شده[31] SS% (متغیر وابسته، کمی پیوسته):

تعریف نظری: SS% مقیاسی سنتی برای اندازه‌گیری شدت لکنت می‌باشد که به این ترتیب محاسبه می‌شود: تعداد هجاهای لکنت شده در نمونه گفتار تقسیم بر کل هجاهای گفته شده در آن نمونه گفتار ضرب در صد [۳۹].

تعریف کاربردی: در این مطالعه مقدار SS% برای تکالیف خواندن با صدای بلند، روایت‌گویی و مکالمه قبل از درمان و نیز یک هفته و ۶ هفته بعد از درمان محاسبه خواهد شد. برای انجام تکلیف خواندن از متون ۱۰۰ کلمه‌ای پیش از درمان، یک هفته پس از درمان و ۶ هفته پس از درمان استفاده خواهد شد.

---

28 Frequency

29 Duration

30 Physical concomitants

31 Percentage of stuttered syllables

-امتیاز پرسشنامه ارزیابی جامع تجربه فرد از لکنت[32] (OASES) (متغیر وابسته، کمی گسسته):

تعریف نظری: این پرسشنامه تجربه فرد مبتلا به لکنت را در چهار بخش بررسی می‌کند. فرد مبتلا به لکنت در مورد اطلاعات کلی از لکنت، واکنش به لکنت، برقراری ارتباط در موقعیت‌های روزمره و کیفیت زندگی به سوالات با استفاده از مقیاس ۵ نمره‌ای پاسخ می‌دهد. نتیجه آزمون به صورت دو مقدار شدت اثر و میزان اثر محاسبه و گزارش می‌شود. در سال ۲۰۱۸ این پرسشنامه به زبان فارسی ترجمه و روایی و پایایی آن برای افراد مبتلا به لکنت فارسی زبان محاسبه شده است [۴۰].

تعریف کاربردی: در مطالعه حاضر برای ارزیابی جامع تجربه فرد از لکنت قبل و پس از درمان، از پرسشنامه فارسی OASES استفاده خواهد شد. همچنین جهت بررسی ماندگاری اثر درمان در تجربه فرد و کیفیت زندگی، ۶ هفته پس از درمان نیز این پرسشنامه توسط افراد شرکت کننده تکمیل خواهد شد. نتایج حاصل از این پرسشنامه به صورت دو مقیاس میزان و شدت اثر گزارش می‌شود. میزان اثر همیشه بین ۲۰ تا ۱۰۰ است و براساس آن شدت اثر بین خفیف تا شدید محاسبه می‌شود.

-موج P300:

تعریف نظری: P300 یک مؤلفه پتانسیل وابسته به رخداد است که بین ۳۰۰ تا ۶۰۰ میلی ثانیه پس از آغاز محرک در پاسخ به محرکهای کم محتمل رخ می‌دهد و اغلب با یک محرک غیرمنتظره[33] استخراج می‌شود [۴۱].

تعریف کاربردی: در این مطالعه با استفاده از تکلیف ادراکی[34] موج P300 استخراج، سپس دامنه[35] و تأخیر[36] آن قبل و بعد از مداخله مقایسه خواهد گردید. تکلیف ادراکی به این ترتیب خواهد بود که تون خالص با فرکانس ۱kHz پخش می‌شود و به صورت تصادفی تغییر فرکانس اعمال می‌شود و فرکانس تون خالص به ۲kHz تغییر داده خواهد شد. نسبت فرکانس معمول به غیرمعمول ۸۰ به ۲۰ خواهد بود و فرد شرکت کننده باید فرکانس غیر معمول را تشخیص دهد و با فشردن کلید صفحه کلید کامپیوتر به آن واکنش نشان دهد.

۵-۵. هدف کلی

هدف کلی این پژوهش تعیین اثر درمان ترکیبی فیدبک شنیداری تأخیری و tDCS بر افزایش روانی گفتار در افراد بزرگسال مبتلا به لکنت می‌باشد.

۵-۶. اهداف اختصاصی (توصیفی و تحلیلی)

اهداف توصیفی:

۱) تعیین میانگین نمره زیرآزمون بسامد هجاهای لکنت شده SSI-4 در گروه مداخله و گروه مقایسه به طور مجزا پیش از درمان.
۲) تعیین میانگین نمره زیرآزمون دیرش لکنت SSI-4 در گروه مداخله و گروه مقایسه به طور مجزا پیش از درمان.
۳) تعیین میانگین نمره زیرآزمون رفتارهای فیزیکی همراه SSI-4 در گروه مداخله و گروه مقایسه به طور مجزا پیش از درمان.
۴) تعیین میانگین نمره کلی آزمون SSI-4 در گروه مداخله و گروه مقایسه به طور مجزا پیش از درمان.
۵) تعیین میانگین میزان اثر کلی و شدت اثر کلی پرسشنامه OASES در گروه مداخله و گروه مقایسه به طور مجزا پیش از درمان.
۶) تعیین میانگین میزان اثر کلی و شدت اثر بخش اطلاعات کلی OASES در گروه مداخله و گروه مقایسه به طور مجزا پیش از درمان.
۷) تعیین میانگین میزان اثر کلی و شدت اثر بخش واکنش به لکنت OASES در گروه مداخله و گروه مقایسه به طور مجزا پیش از درمان.

---

[32] Overall Assessment of the Speaker's Experience of Stuttering
[33] Oddball
[34] Perception
[35] Amplitude
[36] Latency

۱۹	تأخیر موج P300		*		*	*				میلی ثانیه	مشاهده و تفسیر EEG

۱۰-۵. نوع مطالعه

مطالعه حاضر از نوع کارآزمایی بالینی تصادفی از نوع موازی[۳۷] به صورت دو سو کور[۳۸] می‌باشد.

۱۱-۵. جمعیت مورد مطالعه

نمونه معمولاً از اول بخش آورده می‌شود

جامعه مورد مطالعه این پژوهش افراد بزرگسال مبتلا به لکنت خواهند بود.

۱۲-۵. روش نمونه گیری و تعیین حجم نمونه

نمونه‌گیری اولیه به روش در دسترس است و نمونه مورد نظر از میان جمعیت مورد مطالعه و با توجه به معیارهای ورود و خروج انتخاب خواهند شد. سپس شرکت کنندگان با روش تخصیص تصادفی در دو گروه مداخله و مقایسه قرار خواهند گرفت. در بخش روش اجرا، مراحل انجام تخصیص تصادفی به تفصیل بیان شده‌است.

این نمونه از کجا تامین می‌شود؟

معیارهای ورود:

۱) ۱۸ سال به بالا

۲) راست دست بودن

۳) ابتلا به لکنت رشدی با شدت متوسط و شدید

معیارهای خروج:

۱) عدم رضایت فرد به ادامه درمان

۲) حساسیت پوست سر نسبت به تحریک

معیارهای عدم ورود: ؟؟ این معیارها با استفاده از کتب نقل قول از مرجع معدود می‌توانند بیایند.

۴) استفاده از خدمات گفتاردرمانی خارج از محیط پژوهش

۵) مبتلا بودن به صرع، عقب ماندگی ذهنی، کم شنوایی، اختلالات سیستم عصبی مرکزی و اختلالات حرکتی گفتار

۶) مصرف نکردن داروهای ضد تشنج و آرام بخش

معمولاً در بخش روش اجرا می‌آید

حجم نمونه با استفاده از نرم‌افزار GPower، ۵۰ نفر محاسبه شد که ۲۵ نفر به عنوان گروه مداخله و ۲۵ نفر به عنوان گروه مقایسه خواهند بود.

۱۳-۵. روش اجرای طرح

معمولاً اجرای طرح با مراجعه به مراکز نمونه‌گیری شروع می‌شود نه خرید ابزار!

پیش از آغاز مرحله اجرای پژوهش، مقدمات لازم برای این پژوهش انجام خواهد شد. از جمله این مقدمات خرید ابزارهای مناسب شامل هدفون داخل گوشی، ضبط کننده صدا[۳۹]، نرم‌افزار MATLAB و هدست می‌باشد. اقدام دیگر، آماده نمودن شرایط اجرای پژوهش است که در این بخش نرم‌افزار Audator دانلود و سپس با نصب این نرم‌افزار و نرم‌افزار MATLAB بر روی کامپیوتر محل اجرای پژوهش تنظیمات و برنامه‌نویسی لازم جهت اجرای پژوهش انجام خواهد شد. سپس بخش اجرایی اصلی با انتخاب آزمودنی‌های متناسب با هدف مطالعه آغاز خواهد شد.

---

[37] Parallel randomized control trial

[38] Double blind

[39] Voice recorder

پس از انتخاب افراد براساس معیارهای ورود، در مورد روند انجام مطالعه و تحریک عصبی ارائه شده برای افراد شرکت کننده توضیح داده خواهد شد و سپس افرادی که تمایل به شرکت در مطالعه را دارند فرم رضایت نامه را تکمیل خواهند کرد. همچنین به شرکت‌کنندگان توضیح داده می‌شود که افراد شرکت‌کننده به دو گروه مداخله و مقایسه تقسیم خواهند شد و ممکن است فرد در هر یک از این گروه‌ها قرار بگیرد. افراد شرکت کننده در این مطالعه با استفاده از روش تخصیص تصادفی[40] در دو گروه مداخله و مقایسه دسته‌بندی خواهند شد. تخصیص تصادفی به کمک وب سایت www.randomization.com انجام خواهد شد. لیست توسط همکاری تهیه خواهد شد که در روند اجرای پژوهش هیچ نقشی نخواهند داشت. روند کار به این ترتیب است که تعداد گروه‌ها و تعداد کل حجم نمونه در قسمت مشخص در وب سایت وارد خواهد شد و به هر شرکت کننده یک کد اختصاص داده خواهد شد و به طور تصادفی هر کد در گروه A یا B قرار خواهد گرفت. در مرحله بعد جهت مخفی‌سازی فهرست تهیه شده توسط سایت، از استراتژی پاکت‌های دربسته استفاده خواهیم کرد. به این ترتیب که به تعداد حجم نمونه پاکت تهیه می‌شود و بر روی هر پاکت کد فرد شرکت‌کننده یادداشت می‌شود و داخل پاکت براساس لیست حرف A یا B گذاشته می‌شود. پاکت‌های ضخیم استفاده خواهد شد که حرف داخل پاکت قابل مشاهده نباشد. پس از آن لیست ارائه شده توسط سایت معدوم خواهد شد. یک نفر همکار برای تعیین حالت محرک شم یا واقعی دستگاه tDCS در نظر گرفته خواهد شد و فردی که لیست را تهیه کرده است برای وی توضیح خواهد داد که حرف A و B هر کدام نشان‌دهنده چه گروهی است. هنگامی که فرد آزمودنی پاکت را به وی ارائه دهد براساس حرف موجود در پاکت نوع محرک ارائه شده توسط دستگاه را انتخاب خواهد کرد و سپس مراحل بعدی درمان توسط محقق اصلی ادامه خواهد یافت و بدین ترتیب محقق اصلی که اجراکننده جلسات درمانی می‌باشد نسبت به گروهبندی افراد کاملا بی‌اطلاع خواهد بود. همان‌طور که مشخص است افراد شرکت‌کننده در مطالعه و فرد ارائه‌دهنده درمان کاملا نسبت به اینکه فرد در گروه مداخله است یا مقایسه بی‌اطلاع است و در نتیجه این مطالعه از نوع کارآزمایی دوسور کور خواهد بود.

۵-۱۳-۱: مرحله اجرایی اصلی ترکیب DAF و tDCS:

در مطالعه حاضر، برای هر فرد ۹ جلسه برگزار خواهد شد که شامل ۶ جلسه درمانی و ۳ جلسه ارزیابی قبل و بعد از درمان می‌باشد. ارزیابی‌های این مطالعه در دو بخش ارزیابی شدت لکنت و ارزیابی الکتروفیزیولوژیک انجام خواهند شد. جهت انجام ارزیابی‌های مربوط به اندازه‌گیری شدت لکنت پیش از شروع مداخله از مقیاس‌های درصد هجای لکنت شده (SS%)، آزمون SSI-4 و پرسشنامه OASES استفاده خواهد شد، که این مقیاس‌ها در بخش ابزار جمع‌آوری داده‌ها به طور مفصل شرح داده شده‌اند. جهت محاسبه مقیاس SS% از متن صد کلمه‌ای در تکلیف خواندن، و ۱۵ دقیقه روایت‌گویی و مکالمه استفاده خواهد شد. در ارزیابی پیش از مداخله، یک هفته پس از مداخله و ۶ هفته پس از درمان از متون ۱۰۰ کلمه‌ای متفاوت استفاده خواهد شد. به این دلیل متن‌ها متفاوت خواهند بود که اثر تطابق رخ ندهد و اندازه‌گیری شدت لکنت به درستی انجام شود. در مرحله اندازه‌گیری درصد هجاهای لکنت شده (SS%) که پیامد اصلی مطالعه است، دو نفر گفتاردرمانگر که دارای حداقل ۵ سال سابقه کار بالینی می‌باشند، درصد هجاهای لکنت شده را برای هر فرد شرکت‌کننده در هر تکلیف محاسبه خواهند کرد. جهت رعایت کور بودن، این فرد در اجرای سایر مراحل مطالعه نقشی نخواهند داشت و نسبت به گروه‌های مورد مطالعه بی‌اطلاع خواهند بود. این دو نفر به صورت مستقل از هم درصد هجاهای لکنت شده را در نمونه گفتار آزمودنی‌ها محاسبه خواهند کرد سپس با استفاده از شاخص کاپا (ساده یا وزنی) توافق بین دو ارزیاب مورد سنجش قرار خواهد گرفت و گزارش خواهد شد. در صورتی که شاخص به دست آمده نشان‌دهنده وجود توافق بین ارزیاب‌ها باشد، عدد مورد نظر ثبت می‌شود و در صورت عدم توافق از نفر سوم درخواست خواهد شد که SS% را در نمونه‌های گفتاری دارای عدم توافق محاسبه نماید. سابقه کار بالینی نفر سوم از دو نفر ارزیاب قبل بیشتر خواهد بود.

جهت انجام ارزیابی الکتروفیزیولوژیک، پیش از شروع جلسات درمانی ثبت EGG-ERP به عنوان خط پایه[41] انجام خواهد شد. جهت انجام این ثبت از تکلیف ادراکی استفاده خواهد شد. به این ترتیب که محرک تون خالص[42] از طریق هدفون به گوش فرد ارائه خواهد شد و در حین

---

[40] Random allocation

[41] Base line

فرم اعلام اشکالات پروپوزال پایان‌نامه دکتری

دانشجو: خانم نرگس معین

استاد راهنما: دکتر محمدی و دکتر رستمی

استاد داور: دکتر امیری‌شوکی

درج اصلاحات پیشنهادی استاد محترم داور	پاسخ دانشجو قابل قبول است.		ملاحظات
	بلی	خیر	
با سلام و عرض ادب			
و تبریک و آرزوی موفقیت برای جنابعالی			
لطفا اشکالات نگارشی اصلاح شود. مثلا تورفتگی اول پاراگراف			
متغیر اول "تحریک مغز"، آیا واقعا متغیر است؟			
در خصوص جدول متغیرها نظراتی داشتم که چون آقای دکتر کمالی فرمودند و یادداشت فرمودید و بنده نمی‌نویسم.			
آیا با DAF، مغز را تحریک می کنید؟ منظورتان چیست؟	*		
دلیل استفاده از DAF؟ آیا به تنهایی درمان قابل قبولی هست یا چون نمی خواهند درمان شوند ....؟	*		
با حذف ERP موافقم.			
اثر placebo را چه کار می کنید؟ چرا گروه tdcs به تنهایی ندارید؟	*		
مقایسه دو گروه را اضافه بفرمایید.			
جدول صفحه ۱۸ و ۱۹ یکجا باشد.			
منابع را انگلیسی پاراگراف‌بندی نمایید.			

نام داور:

امضا:
